# A taxonomic synopsis of Altingiaceae with nine new combinations

**DOI:** 10.3897/phytokeys.31.6251

**Published:** 2013-12-17

**Authors:** Stefanie M. Ickert-Bond, Jun Wen

**Affiliations:** 1 UA Museum of the North Herbarium (ALA), Department of Biology and Wildlife, University of Alaska Fairbanks, 907 Yukon Drive, Fairbanks, Alaska 99775-6590 USA; 2 Department of Botany, MRC-166, Smithsonian Institution, P.O. Box 37012, Washington, D.C. 20013-7012 USA

**Keywords:** *Altingia*, Altingiaceae, *Liquidambar*, *Semiliquidambar*, taxonomic synopsis

## Abstract

A taxonomic synopsis of the Altingiaceae is presented, including the taxonomic enumeration and distribution of 15 recognized species based on studies of 1,500 specimens from 24 herbaria throughout the distributional range of the taxa. Previous phylogenetic analyses based on several molecular markers have shown that *Altingia* and *Semiliquidambar* are nested within *Liquidambar*. All *Altingia* and *Semiliquidambar* species are now formally transferred to *Liquidambar*, which has the nomenclatural priority. The following nine new combinations are herein made: *Liquidambar cambodiana*(Lecomte) Ickert-Bond & J. Wen, *Liquidambar caudata* (H. T. Chang) Ickert-Bond & J. Wen, *Liquidambar chingii* (Metcalf) Ickert-Bond & J. Wen, *Liquidambar gracilipes* (Hemsl.) Ickert-Bond & J. Wen, *Liquidambar multinervis*(Cheng) Ickert-Bond & J. Wen, *Liquidambar obovata* (Merrill & Chun) Ickert-Bond & J. Wen, *Liquidambar poilanei* (Tardieu) Ickert-Bond & J. Wen, *Liquidambar siamensis* (Craib) Ickert-Bond & J. Wen, and *Liquidambar yunnanensis* (Rehder & Wilson) Ickert-Bond & J. Wen.

## Introduction

The Altingiaceae (the sweet-gum family) are a small family of trees that have been traditionally classified into members with a predominantly temperate distribution (*Liquidambar* L.) and those with a largely tropical to subtropical distribution (*Altingia* Noronha, *Semiliquidambar* H. T. Chang). The family is valued worldwide for its timber and fragrant resin (styrax) and is locally highly prized for the roots and bark used in traditional Chinese medicine (Vink 1957; Zhang et al. 2003; Ickert-Bond et al. 2007). Most noteworthy to biologists, Altingiaceae show a fascinating intercontinental disjunction in temperate regions of North America, W Asia and some higher elevational montane areas in subtropical Asia and Mexico (Ickert-Bond and Wen 2006). Furthermore, deep molecular divergence coupled with a high level of morphological similarity suggests a conserved morphology of some species, i.e., morphological stasis, an evolutionary phenomenon that has been proposed for many animal groups as well as some plants (Ickert-Bond and Wen 2006). Other members of the Altingiaceae exhibit morphological divergence in response to habitat diversity in the subtropics of eastern Asia. One member of the sweet-gum family, *Semiliquidambar*, has puzzled scientists since its discovery in the 1960s, due to its rarity and morphological intermediacy between the other two genera (Ferguson 1989).

Generally, the Altingiaceae were considered closely related to the Hamamelidaceae (see below). The family is recognized by the solitary capitate woody infructescences with many bicarpellate fruits, and male inflorescences in heads aggregating into racemes. Other systematic characters that differentiate the two families are less well known and understood. It is now generally agreed that the Altingiaceae are a distinct family (Magallón et al. 1999); for detailed comparisons see our other contributions (APG II 2003; Pigg et al. 2004; Ickert-Bond et al. 2005, 2007; Ickert-Bond and Wen 2006; APG III 2009).

The family name Altingiaceae is based on *Altingia*, first named by Noronha (1790) in honor of the former General Governor Alting of the East Indian colonies of the Netherlands (Hayne 1830). The family was formally designated in 1843 by Horaninow (Hoogland and Reveal 2005). The scientific name for *Liquidambar* L. is a combination of the Latin and Arabic words *Liquidus* and *Amber* meaning fragrant liquid or balsam (Yaltrik and Efe 2000). Most authors have recognized *Altingia* and *Liquidambar* to be members of the Hamamelidaceae s.l., most often at the subfamily level, while Endlicher in his *Genera Plantarum* (1840) segregated the Altingiaceae [s. Balsamifluae] from the Hamamelidaceae s. str and placed Altingiaceae in *Juliflorae*, between Platanaceae and Salicaceae, while the Hamamelidaceae were placed in *Discanthae*, between Loranthaceae and Bruniaceae. Subfamily Altingioideae was recognized by J. Williams in his revision of Balfour’s Manual of Botany in 1855. Bentham and Hooker (1883) treated *Liquidambar* and *Altingia* as distinct genera alongside other typical genera of Hamamelidaceae, without mention of subfamilies, but they recognized two categories (Abteilungen): (1) with the ovary containing 2- many ovaries, and (2) with the ovary containing a single ovary. Reinsch (1890) retained the traditional one family concept, but departed from the general consensus by splitting the Hamamelidaceae based on morphological and anatomical characters, that he considered to be more fundamental than the fruit, into three subfamilies: (1) Altingioideae including *Altingia* and *Liquidambar* and (2) Bucklandioideae including *Exbucklandia* R.W. Brown and *Rhodoleia* Champ. ex Hook., and (3) Hamamelidoideae including *Corylopsis* Siebold & Zucc.,* Dicoryphe* Thouars,* Distylium* Siebold & Zucc., *Eustigma* Gardner & Champ.,* Fothergilla* L.,* Hamamelis *L.,* Loropetalum* R. Br.,* Parrotia* C.A. Mey.,* Sycopsis* Oliv. and *Trichocladus* Pers., (*Semiliquidambar* was not known at the time).

Baillon (1871) explicitly excluded *Liquidambar* and *Altingia* from the Hamamelidaceae, contrary to Bentham and Hooker, who included these two genera in the family. Baillon placed them in an intermediate position between Hamamelidaceae and Platanaceae. Traditionally, the Hamamelidaceae s.l. (including Altingiaceae) have been considered as a member of the Hamamelididae Takht. (Cronquist 1981; Takhtajan 1997). Recent molecular studies have shown this assemblage to be polyphyletic and support Altingiaceae and Hamamelidaceae s. str. as members of the saxifragoid clade within a larger rosid clade (Chase et al. 1993; Magallón et al. 1999; Soltis et al. 2000; APG II 2003; Fishbein and Soltis 2004; Soltis et al. 2007; APG III 2009; Soltis et al. 2011). Furthermore, relationships among some Saxifragales, and the remaining families (Altingiaceae, Cercidiphyllaceae, Daphniphyllaceae, Hamamelidaceae, and Paeoniaceae) also remain unclear (Feng et al. 1998; Qiu et al. 1998; Hoot et al. 1999; Savolainen et al. 2000; Fishbein et al. 2001; Soltis et al. 2000). In maximum likelihood analyses of a five-gene data set, Fishbein et al. (2001) recovered an optimal topology with Daphniphyllaceae and Hamamelidaceae sister to the remaining members of the clade; however, the precise branching order of these two early-diverging members of Saxifragales was unclear. Following Daphniphyllaceae and Hamamelidaceae, Altingiaceae, Cercidiphyllaceae, and Paeoniaceae appeared as successive sisters to a core clade of Saxifragaceae, Haloragaceae and Crassulaceae. Fishbein et al. (2001) also showed that the poor resolution obtained in Saxifragales is not due to violations of assumptions or to combining data partitions having conflicting histories or processes. Rather, their analyses suggest instead that the initial diversification of Saxifragales was indeed rapid. Within Saxifragales molecular phylogenetic results have rarely supported a sister relationship between Altingiaceae and Hamamelidaceae s. str. (e.g., Hoot et al. 1999; Fishbein et al. 2001, but see Fishbein and Soltis 2004). Most recently, based on over 50,000 bp Jian et al. (2008) have found strong support for a clade composed of the Paeoniaceae + woody clade (Cerdiphyllaceae, Daphnipyllaceae, and Hamelidaceae) Altingiaceae)) to be sister to the rest of the Saxifragales. The sister group relationship of Altingiaceae with Hamamelidaceae plus Cerdiphyllaceae and Daphniphyllaceae was also strongly supported in a supermatrix approach by Soltis et al. (2013).

*Altingia* and *Liquidambar* are each defined by several morphological characters and have been maintained as separate genera in modern taxonomic treatments (Vink 1957; Tardieu-Blot 1965; Zhang et al. 2003). Analyses based on several molecular markers suggest that *Altingia* is nested within *Liquidambar* (Shi et al. 1998; Shi et al. 2001; Ickert-Bond et al. 2005; Ickert-Bond and Wen 2006; Ickert-Bond et al. 2007, Wu et al. 2010) and that *Semiliquidambar* is of intergeneric hybrid origin between *Liquidambar formosana*–*Liquidambar acalycina* and *Altingia obovata* or *Altingia chinensis*. Yet our morphological analysis supports *Altingia* and *Liquidambar* as mutually exclusive sister clades (Ickert-Bond et al. 2005, 2007). The apparent incongruence of these phylogenies appears to be due to morphological convergence.

Characters that distinguish *Liquidambar* from *Altingia* are related to an open wind pollination syndrome and may represent convergences to temperate habitats, particularly, the presence of anthers borne on long filaments and the loss of stomium bifurcations would facilitate the wind dispersal of pollen (Hufford and Endress 1989), while long narrow styles on exserted fruits (Fig. 2D) may aid in the capture of pollen on the broad stigmatic surfaces in open habitats of temperate *Liquidambar*. Furthermore, additional synapomorphies for *Liquidambar* may also represent adaptations for a temperate distribution. These characters (elongate and tapered carpel shape, seeds with distal wings, and more tightly constructed infructescences) are related to seed rather than pollen dispersal. Several other families (e.g., Platanaceae) show a similar convergence among temperate members (Tiffney 1986; Crane 1989).

Character-state changes in *Altingia* seem to correlate with tropical and subtropical environments in eastern Asia and Indochina, whereas changes in *Liquidambar* correlate with temperate sites, where the genus is found today. Of the eight characters defining *Altingia* (Fig. 2A, B), four are reversals (characters 2–5: ratio of leaf length to width, leaf division, venation, and stipule size) (see fig. 86 of Ickert-Bond et al. 2007). The availability of diverse habitats in tropical and subtropical eastern Asia and Indochina facilitated the diversification of *Altingia* species in response to recent active uplifts of mountains in eastern Asia since the Tertiary (Morley 1999; Wen 1999, 2001; Ickert-Bond and Wen 2006).

To maintain the monophyly of the group in question (Potter and Freudenstein 2005), we place all taxa of Altingiaceae in *Liquidambar* (the earliest available name), and maintain the conserved name Altingiaceae for the family. Appropriate new combinations are provided below.

**Figure 1.**
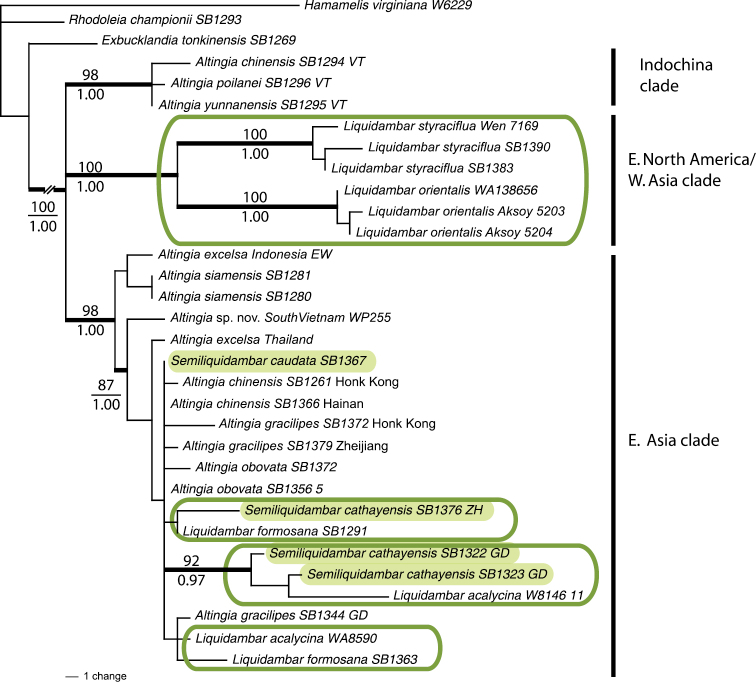
Phylogenetic relationships of Altingiaceae based on maximum likelihood analyses of combined *cp*DNA data. Phylogram is one of 14 trees (-In*L*=9927.72) derived from maximum likelihood analyses showing rates of substitution under K81uf+I model of substitution evolution (Modified from Ickert-Bond and Wen 2006). Note: Break in branch lengths for the OG at left, and *Semiliquidambar* taxa with green shaded boxes, clades that include *Liquidambar* taxa have green outline boxes.

**Figure 2.**
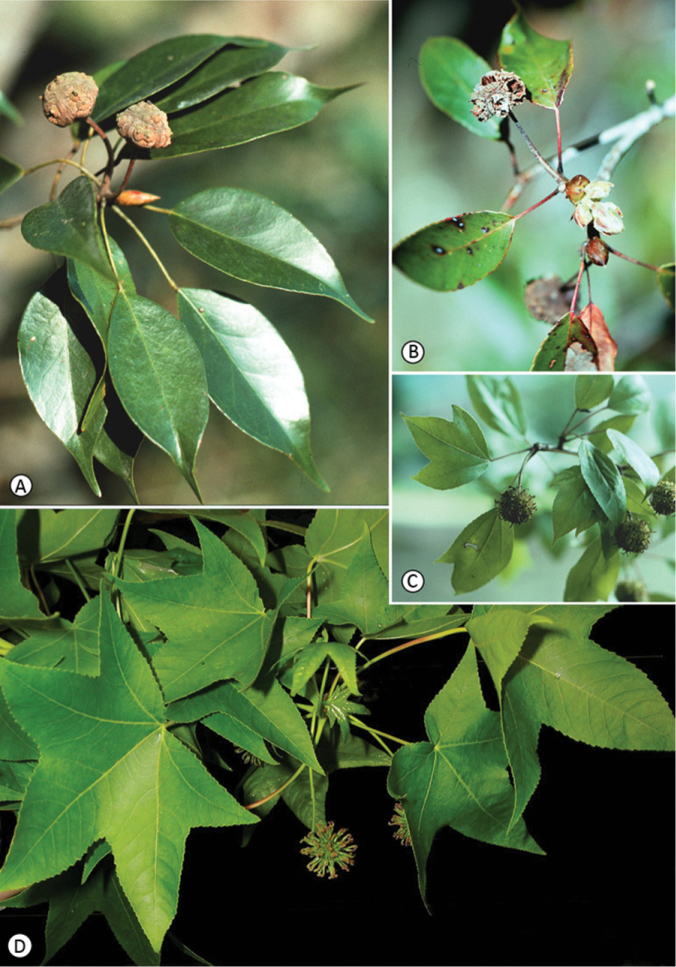
Morphological variation in Altingiaceae. **A** *Liquidambar gracilipes* with obconical flattened infrutescences and short styles. Leaves are simple, entire, choriaceous, and show a characteristic drip tip at the apex **B** *Liquidambar siamensis* also shows obconical flattened infructescences with simple leaves that have a serrate margin and lack of a drip tip **C** *Liquidambar chingii* showing variation in leaf morphology from palmately three-lobed leaves at left, to two-lobed leaves and simple leaves on the same branch. Infructescences are globose with long styles **D** *Liquidambar styraciflua* showing palmately five-lobed, chartaceous leaves, and globose infructescences with long styles.

## Methods

We evaluated all currently recognized taxa within *Altingia*, *Liquidambar*,and *Semiliquidambar*. Our study is based on: (1) field observations from throughout the distributional range of the taxa, with field visits to sites in Mexico (Veracruz), Vietnam, Cambodia, Indonesia and Guangdong, Hainan, Hong Kong, Hubei, Giangxi, and Zheijang provinces in China, and (2) the analysis of specimens in 24 herbaria (including available types): A, BK, BM, C, E, F, FI, FN, FUS, GH, HGAS, HN, IBSC, ISTO, K, LINN, LU, N, NFU, NY, P, PE, SYS, US. We previously provided detailed examination of the fruit anatomy and morphology (Pigg et al. 2004; Ickert-Bond et al. 2005; Ickert-Bond et al. 2006, 2007) and here provide an overview of some of the features that have been used to characterize the genera both for ovulate and staminate infrutescences. Measurements were made with an electronic caliper (Mitutoyo mod. CD-6”CS).

Pollen of selected species from all three genera were studied to assess the taxonomic utility in this group. Pollen samples were obtained from herbarium material deposited at F unless otherwise noted (*Liquidambar chinensis* [*S. Ickert-Bond 1319]*; *Liquidambar excelsa* [*Widjaja* s.n.], *Liquidambar gracilipes* [*S. Ickert-Bond 1344*], *Liquidambar obovata* [*Wang 36153*], *Liquidambar poilanei* [*S. Ickert-Bond 1296*], *Liquidambar siamensis* [*S. Ickert-Bond 1281*], *Liquidambar acalycina* [Chui 3191 (MO)], *Liquidambar formosana* [*C. Tan 93025* (2 sheets, MO)], *Liquidambar styraciflua* [*Vazquez T*. 153], *Liquidambar chingii* [*S. Ickert-Bond 1330*, *S. Ickert-Bond 1320*], and acetolyzed (Erdtman 1960), OTOTO coated (Kelley et al. 1973; Chissoe et al. 1994, 1995), freeze fractured (Skvarla et al. 1988), dried with HMDS (Nation 1983; Chissoe et al. 1994), mounted on stubs with double-sided tape, coated with approximately 200 Å of gold in a Denton Vacuum Desk II vacuum evaporator or sputter coated with a gold/palladium target (60/40) in a Hummer VI Sputter Coating System (Chissoe and Skvarla 1996), and viewed with a JEOL JSM-880 scanning electron microscope (SEM) at 10–15 kV.

## Data resources

The data underpinning the analyses reported in this paper are deposited at GBIF, the Global Biodiversity Information Facility, http://ipt.pensoft.net/ipt/resource.do?r=altingiaceae_synopsis .

## Taxonomic treatment

**Altingiaceae** Horan., Osnov. Bot.: 271. 1841, nom. conserv. TYPE: *Altingia* Noronha, 1790. – Validated by a reference to an effectively, but not validly published Blume & J. Fischer (Fl. Javae 17-18: 3. 1829, as Balsamifluae) family name with a description in Latin and proposed as an alternative name. – Isonyms: Horaninov, Tetracytys: 25. 1843 (“Altingiaceae (s. Balsamifluae”), validated by a reference to Blume & J. Fischer (1829); see also Lindley, Veg. Kingdom: 253. 14–28 Mar 1846, validated by a description in English). The earlier Hayne (Flora 13: 172. 1830) name is a nom. nud.

Trees, deciduous or evergreen; terminal buds perulate, narrowly ovoid. Leaves petiolate; stipules usually present, linear, ± adnate to base of petioles, caducous, leaving small scars; leaf blade palmately 3–7(or more)-lobed, or if entire lanceolate to ovate or obovate, leathery, discolorous, margin usually crenate-serrate, occasionally entire, venation pinnate or leaf blade palmately 3–7(or more)-lobed, venation actinodromous. Plants monoecious. Male inflorescence a globose to shortly cylindrical, pedunculate, many-flowered head, grouped in terminal or subterminal, compound racemes or panicles; each flower with 1–4 basal bracts. Female inflorescences capitate, subterminal or in lower part of male inflorescence, long-pedunculate, 5–30-flowered. Flowers unisexual. Sepals and petals absent. Male flowers: stamens (4–) many; filaments very short or absent; anthers obovate-ovoid, thecae 2-sporangiate, each dehiscing by a longitudinal slit or rudimentary valve, apex truncate; pollen spheroidal, polyporate. Female flowers: staminodes (also interpreted as carpellodes) absent or needlelike; ovary semi-inferior; ovules ca. 30–50 per locule, axile; styles subulate, divergent, often strongly recurved; stigmas papillose, basal parts or whole styles persistent in fruit. Infructescences globose, base truncate. Capsules woody, dehiscing loculicidally by two 2-lobed valves, also septicidally; staminode teeth and styles not persistent. Seeds many, upper ones sterile, one or a few lower ones fertile, flattened, narrowly winged along margin or only at apex; seed coat thick and hard; endosperm thin. 2*n* = 32.

One genus and ca. 15 species: E, W, and SE Asia, Central, and North America.

**Figure 3.**
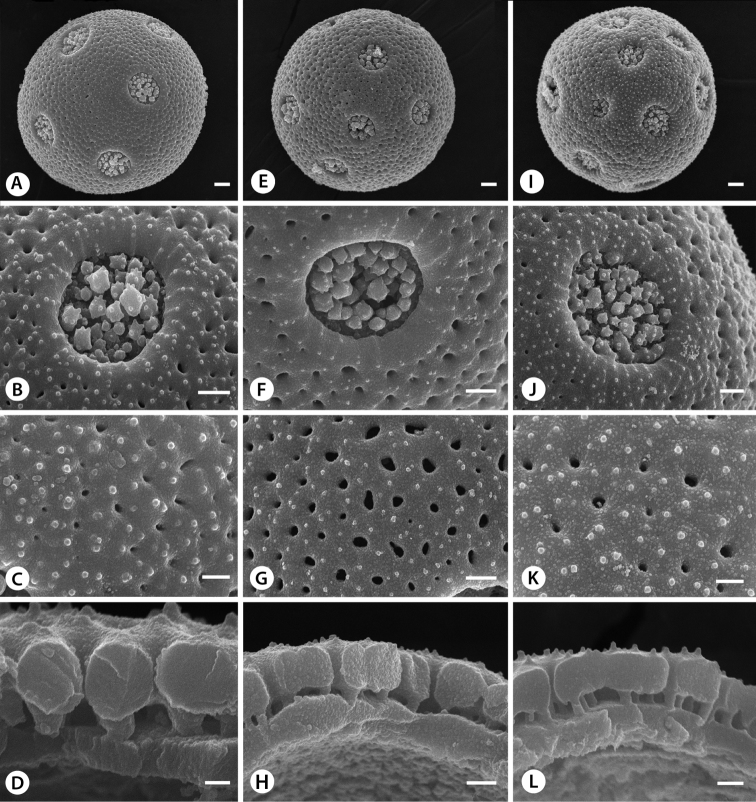
Pollen diversity in Altingiaceae. **A–D** *Liquidambar excelsa* **E–H** *Liquidambar gracilipes* **I–L** *Liquidambar styraciflua* **A** Pentaporate, spheroidal pollen grain **B** Details of pore showing the characteristic disintegration of the tectum **C** Details of the tectal surface displaying scabrae and irregularly-shaped perforations **D** Details of tectate-columellate exine, note columellae slightly thicker than those of comparable magnification in **H** and **E** Pentaporate, spheroidal pollen grain **F** Details of pore showing the characteristic disintegration of the tectum **G** Details of the tectal surface displaying scabrae and irregularly-shaped perforations **H** Details of tectate-columellate exine with thinner columellae than in **D** of *Liquidambar excelsa* **I** Pentaporate, spherical pollen grain **J** Details of circular pore showing eroded tectum in the pore proper **K** Details of tectal surface with irregularly-shaped perforations and scabrae **L** Details of the tectate-columellate exine with thin columellae as compared to *Liquidambar excelsa* in **D**. Scale bars: **A, E, I** = 2 µm, scale bars: **B, F, G, J** = 1 µm, scale bars: **C, H, K, L**= 500 nm, scale bar: **D**= 200 nm.

Pollen morphology appears uniform throughout the family with spherical, pentaporate grains (Fig. 3A, E, I), that show scabrae and irregularly shaped perforations on the tectum (Fig. 3B–C, F–G, J–K) and a tectate-columellate exine (Fig. 3D, H, L; Ferguson 1989). Zavada and Dilcher (1989) found slight differences in the breadth of the columellae between *Liquidambar styraciflua* and *Altingia obovata* based on TEM imagery. Our analysis of the exine using freeze-fracturing and SEM shows slight difference of this characters between *Liquidambar excelsa* (= *Altingia excelsa*, Fig. 3D) and *Liquidambar styraciflua* (Fig. 3L), but the columellae in *Liquidambar gracilipes* (= *Altingia gracilipes*, Fig. 3H) appear to be of equal width to those in the ones examined from *Liquidambar styraciflua* (Fig. 3L).

### Key to the species of *Liquidambar* s.l.*

**Table:** 

1	Leaves consistently palmately-lobed
2	Leaves of mature trees with three lobes, sometimes 5 in juvenile condition
3	Infructescence subglobose, with stout styles 4–6 mm long, curved; seeds with circular flange	1. *Liquidambar acalycina*
3’	Infructescences globose, with fine styles 7–10 mm long, coiled; seeds with a terminal wing	7. *Liquidambar formosana*
2’	Leaves of mature trees with more than 3, mostly 5 lobes, sometimes more than 5 lobes present
4	Infructescences with narrow style bases (up to 18 mm wide); areas between fruits appearing as a smooth rim	11. *Liquidambar orientalis*
4’	Infrutescences with broad style bases (up to 30 mm wide); areas between adjacent fruits appearing braided	14. *Liquidambar styraciflua*
1’	Leaves variable, mostly unlobed or varying to lobed within a single branch
5	Leaves tri-nerved, mostly palmately-lobed, seldom unlobed	5. *Liquidambar chingii*
5’	Leaves penninerved, strictly unlobed
6	Infructescences obconical with 5-8 fruits, base of the infructrescence with a prominent “skirt”
7	Leaves coriaceous, 2–3 cm wide, glossy above; margin entire, less often serrate; fruits up to 2 cm broad	8. *Liquidambar gracilipes*
7’	Leaves chartaceous, 3–4 cm wide, dull above; margin serrate; fruits broader than 3 cm	13. *Liquidambar siamensis*
6’	Infructescences mostly subglobose, with 10–30 fruits, base of the infructescence lacking a “skirt”
8	Leaves glossy above, margins distinctly revolute upon drying; endemic to Cambodia	2. *Liquidambar cambodiana*
8’	Leaves dull above, margins not curled; of broader distribution in southeast Asia
9	Petioles 0.5–1.2 cm long
10	Leaves elliptical	4. *Liquidambar chinensis*
10’	Leaves obovate
11	Leaf base acute, apex obtuse, lateral veins 8–10, conspicuous beneath	10. *Liquidambar obovata*
11’	Leaf base subcordate to rounded, base acute, lateral veins 5–6, not prominent beneath	12. *Liquidambar poilanei*
9’	Petioles 2–5 cm long
12	Leaves chartaceous	6. *Liquidambar excelsa*
12’	Leaves coriaceous
13	Leaves 4–7 cm long, apex caudate, petioles slender, 1–1.5 mm thick; infructescences 1–2 cm broad	3. *Liquidambar caudata*
13’	Leaves 8–13 cm long, apex acute, petioles stout, 2–3 mm thick; infructescences 2–3 cm broad	15. *Liquidambar yunnanensis*

* *Liquidambar multinervis* is poorly understood at present and is not included in the key.

#### Liquidambar

L.

http://species-id.net/wiki/Liquidambar

in Sp. Pl. 2:999. 1753.

Altingia Noronha, in Verh. Bal. Gen. v. (1785) Art. ii. 9; ed. II. 41, nom. cons., syn. nov.
Semiliquidambar H. T. Chang, in Acta Sct. Nat. Sunyatseni: 35, 1962, syn. nov.

##### Type.

*Liquidambar styraciflua* L.

#### 1. Liquidambar acalycina

H. T. Chang

http://species-id.net/wiki/Liquidambar_acalycina

Acta Sci. Nat. Univ. Sunyatseni, 1959 (2): 33. 1959. Type. CHINA: Hunan: Wugang, *H. T. Chang 4701* (holotype: IBSC 001053!).

##### distribution.

China (Anhui, Guangdong, Guangxi, Guizhou, Hubei, Hunan, Jiangsu, Jiangxi, Sichuan, and Zheijiang). **Representative specimens examined**. **China**: **Fujian**: Wuyi Mountain, *Wuyi Shan Team 80-225* (MO);Wuyishan, Hengkeng, *H.-Y. Zou 1984* (MO); **Guangdong**: Ruyuan Xian, *C. Wang 44102* (MO); **Guangxi:** Damiaoshan, Shanfang Qu, Cidong Xian, Jiuwanshan, *S.-Q. Chen 14715* (MO); **Guizhou**: Yinjiang Xian, along the trail between Zhangjiaba and Huguoshi on the west side of the Fanjing Shan mountain range, *B. Bartholomew 1660* (GH); Yinjiang Xian, vicinity of Xiapingsho on the west side of the Fanjing Shan mountain range, *B. Bartholomew 1758* (GH, MO);**Hubei**: Lichuan, *Metasequoia* Region of Lichuan Xian (Hsien), vicinity of Lojiaba on the W side of the valley, *B. Bartholomew 1950* (GH); no locality, *E.H. Wilson 513* (GH); vicinity of Shui-sa-pa, *J.L. Gressitt 2415* (GH); **Hunan**: Henyuang, Goulowshan, *J.B. Zuo 356* (MO); Hengshan, Nanyue, Longci, *S.-Q. Chen 3346* (2 sheets, MO); Yizhang, Mangshan, Datangken, *L.-H. Liu 542* (MO); **Jiangxi**: De-Xin, De-xin county, *K. Yao 11486* (GH); Lushan, *M.L. Nie 91192* (MO); **Sichuan:** S. Wushan, *A. Henry 52181* (GH); **Zheijiang**: Linan Xian, Changhua, Bailongtanshan, *P.L. Chiu 961* (MO).

##### Cultivated.

Arnold Arboretum, grown from seeds collected from Hubei province during the Sino-American Botanical Expedition (*SABE 1950* [GH]) near Lojiaba in the *Metasequioa* Valley of Lichuan Xian in October 1980.

#### 2. Liquidambar cambodiana

(Lecomte) Ickert-Bond & J. Wen comb. nov.

urn:lsid:ipni.org:names:77134706-1

http://species-id.net/wiki/Liquidambar_cambodiana

Basionym: *Altingia cambodiana* Lecomte, Bull. Mus. Hist. Nat. Paris 30: 391 (1924). TYPE. CAMBODIA: Mont d’Éléphant, Se mean phnom, *Poilane 263* (holotype: P [P00749065!]; isotype: P [P003173561!]).

##### Note.

A unique species with only three specimens from one locality in Cambodia known. The protologue does not include reference to where *Poilane 263* is deposited. Furthermore, the two specimens at P are the only ones that include both “*Poilane 263*” and the locality information on the sheet (agreeing with the type description). The other two sheets, one at the Smithsonian (US 150518!) and one at the Edinburgh Botanic Gardens Herbarium (E 00181744!) include only labels that state *Herb. Mus. Paris, Altingia cambodiana H. Lec. Institute Scientifique de Saigon – M. Poilane Reçu le 20 May 1921*. These two later sheets (US 150518! And E 00181744!) most likely were part of the original material that Lecomte saw when he described *Altingia cambodiana*.

##### Distribution.

SW Cambodia. **Representative specimens examined**. **CAMBODIA**.* M. Poilane s.n*. (US 150518!), (E 00181744!).

#### 3. Liquidambar caudata

(H. T. Chang) Ickert-Bond & J. Wen comb. nov.

urn:lsid:ipni.org:names:77134707-1

http://species-id.net/wiki/Liquidambar_caudata

Basionym: *Semiliquidambar caudata* H. T. Chang, Acta Sci. Nat. Univ. Sunyatseni, 1962: 39. 1962. TYPE. CHINA: Fujian: Shaxian County, *FUS expedition 53260* (holotype; FUS 47396- seen as photo!).

Semiliquidambar cuspidata H. T. Chang, Acta Sci. Nat. Univ. Sunyatseni, 1962: 39, 1962. TYPE. CHINA: Zhejiang: Jingning, *Hangzhou Botanial Garden 7303* (holotype: PE [PE1392499!]; isotype: MO [MO4525923!].

##### Note.

This taxon is similar to *Altingia gracilipes*, but its leaves are not strongly 3-nerved at the base.

##### Distribution.

Fujian and Zhejiang provinces of China. **Representative specimens cited**. **China: Zhejiang:** Ye Ling, Tai Shun County, *Z.G. Mao 10237* (MO 4491051); Ying Chuan, Jing Ning county, *S.Y. Chang 4837* (MO 4491052, MO 4536933).

#### 4. Liquidambar chinensis

http://species-id.net/wiki/Liquidambar_chinensis

Champ., Kew Journ. Bot. 4: 164, 1852. TYPE. CHINA: Hong Kong, Champion 325 (lectotype here designated: K [H2007/01764!]).

Altingia chinensis (Champ.) Oliver ex Hance, in J. Linn. Soc. 13: 103, 1873.
Altingia chinensis f. *pubescens* X.H. Song, in J. Nanjing Inst. Forest 4: 49, 1984. TYPE: CHINA: Guizhou: Libo, *X.H. Song 1348* (holoype: NFU!).

##### Note.

No specimen was mentioned in the type description. *Champion 325* (K) is the only Champion specimen of *Altingia* found by us so far. It bears a label with a handwritten identification *Liquidambar* sp. nov. by Champion (based on comparison with other holotype material described by Champion at K). This specimen is thus designated as the lectotype for *Liquidambar chinensis* Champ.

##### Distribution.

China (Guangdong, Guangxi, Guizhou, Hainan, Hong Kong), Vietnam. **Representative specimens examined.** **CHINA:** **Guangdong**: Kwai Shan, Tsing-lo-kong village, Ho-yuen district., *W.T. Tsang 28544* (A); Lin Fa Shan, Sam Hang Shek T’au Village, Hwei-yang District, *W.T. Tsang 25942* (A, E); Naam Kwan Shan, Tsengshing District, *W.T. Tsang 20218* (E, MO); Poon Yue district, *C.O. Levine 3158* (MO); Xinyi Xian, *C. Wang 31828* (MO); **Guangxi:** Chen Pien District, *S.P. Ko 56024* (A); Foo Lung, Sup Man Ta Shan, *H.Y. Liang 69714* (A); Pingnan Xian, *C. Wang 39334* (MO); Shap Man Taai Shan, near Hoh Lung village, SE of Shang-ze, Guangdong border (Shang-ze district), *W.T. Tsang 22577* (A); Shap Man Taai Shan, near Iu Shan village, SE of Shang-ze, Guangdong border, Shangze district, *W.T. Tsang 22189* (F); She-Feng Dar Shan, S. Nanning, *R.-C. Ching 7937* (A); Tong Shan (along Guangdong border), near Sap-luk Po village (Waitsap district), *W.T. Tsang 22788* (A); **Hong Kong**: Aberdeen Rd., H.C. Tang 590 (HK), *Y.S. Lau 206* (HK); Jardin Botanique, *E. Bodinier 1042* (E); Hong Kong Botanical Garden, *S. Ickert-Bond 1274* (F); Lokchong, *C.L. Tso 21049* (F); Luk Keng, Shek Pan Tam, *P.-S. Choi s.n*. (HK); Ma On Shan, *K.E. Wong s.n*. (HK); Shing Mun Country Park, Shing Mun Arboretum, *S. Ickert-Bond 1261* (F); **Zhejiang**: Feng Yang Mtn., *H.-Y. Zou 307* (A),* H.-Y. Zou 761* (MO). **VIETNAM: Lao Cai**: SaPa, *A. Petelot 2332* (MO); *M. Brillet 19* (P, 2 sheets).

#### 5. Liquidambar chingii

(Metcalf) Ickert-Bond & J. Wen comb. nov.

urn:lsid:ipni.org:names:77134708-1

http://species-id.net/wiki/Liquidambar_chingii

Basionym: *Altingia chingii* Metcalf, Lingnan Sc. Journ. 10: 413, 1931. TYPE. CHINA: Fujian: near Zhejiang border, 2800 ft., *R.-C. Ching 2244* (holotype: SYS [SYS53818!]; isotypes: A [A 00043389!], IBSC [IBSC 001070!), NY [NY 00356121!], PE [PE 217977!]), SYS [SYS000957151!]).

Altingia chingii Metcalf var. *parvifolia* Chun, Sunyatsenia 1: 241, 1934. *Semiliquidambar cathayensis* H. T. Chang var. *parvifolia* (Chun) H. T. Chang, in Acta Sct. Nat. Sunyatseni: 38, 1962. TYPE: CHINA: Guangdong, Ying Tak, Wentong Shan, in mixed woods, *H.Y. Liang 61283* (holotype: IBSC [IBSC001069!]; isotype: PE [PE 512712!]).
Semiliquidambar cathayensis H. T. Chang, Acta Sunyatseni: 37, 1962. TYPE. CHINA: Guangdong: Ruyuan, *S.P. Ko 53448* (holotype: IBSC [IBSC001068!]; isotype: PE [PE 509073!].
Semiliquidambar cathayensis H. T. Chang var. *fukienensis* H. T. Chang, in Acta Sct. Nat. Sunyatseni: 38, 1962. TYPE. CHINA: Fujian: Zhangping, *Y. Ling 4522* (holotype: PE [PE419004!]; isotype: PE [PE83570!].
Semiliquidambar chingii (Metcalf ) H. T. Chang, in Acta Sct. Nat. Sunyatseni: 38, 1962.
Semiliquidambar chingii (Metcalf) H. T. Chang var. *longipes* Y. K. Li & X. M. Wang, in Acta Botanica Yunnanica 8: 275,1962. TYPE. CHINA: Guizhou: Lipo Xian, Wujiahe, *Exped. of Guizhou Academy of Science 76536* (holotype: HGAS!; isotype: SYS [SYS00072758!]).
Semiliquidambar coriacea H. T. Chang, in Acta Sct. Nat. Sunyatseni: 39, 1962. TYPE. CHINA: Guandong: Ruyuan, *C. Wang et C.Y. Li 44074* (holotype: SYS [SYS123416!]; isotype: IBSC [IBSC001071!], PE [PE723787!]).

##### Distribution.

China (Fujian, Guangdong, Guangxi, Guizhou, Jiangxi) and Vietnam (Ha Giang, Lao Cai). **Representative specimens examined**. **CHINA: Guangdong**: Da Ling Tan, Shan Shuai, Li Shan, *Peixiang Tan 58681* (A); Hua Nan Agriculture University, specimen plant garden, Guangzhou, *Zhiming Wu 85062* (MO); Nanling National Forest Park,*S. Ickert-Bond 1307* (F),* 1308* (F),* 1310* (F),* 1311* (F); Zhong Ba Shui, Zhong Ba, Yao Zu county, *L. Deng 6681* (MO); **Hainan**: Bai Shui Ling, Dali County, Dali Mountains, Chengpo district, Qiungzhou County, *L. Deng 3549* (MO); Qiongshong, Ying Ge Ling, eastern slope, *National Geographic Society Hainan Expedition 46* (MO). **VIETNAM:** **Ha Giang**: Vi-Xuyen District, Minh-Tan community, *H. Ngoc Kinh 169* (HN).

#### 6. Liquidambar excelsa

(Noronha) Oken

http://species-id.net/wiki/Liquidambar_excelsa

Allg. Naturgesch. iii. (3) 1539, 1841. Basionym: *Altingia excelsa* Noronha, Verh. Batav. Genootsch. Kunsten. 5(2): 9, 1790.

Altingia caerulea Poir., Encycl. [J. Lamarck & al.] Suppl. 5: 545, 1819.
Liquidambar rasamala Blume, Cat. Gew. Buitenz. 6, 1823.
Liquidambar altingiana Blume, in Flora Javae 17: 8, tt. 1,2, 1829.
Liquidambar cerasifolia (Wall. & Griff.) Voigt, Hort. Suburb. Calcutt. 301. 1845.

##### Distribution.

China, Bhutan, India, Indonesia (Java, Bali, Sumatra), Malaya, Myanmar, and Thailand. **Representative specimens examined**. **BHUTAN.** Sarbhang district: above Noonpani, 16 km along Sarbhang-Chirang road, *A. Grieson 3581* (E). **CHINA. Yunnan**: between Muang Hing and Szemao and the Szemao hills proper, Southern Yunnan, *J.F. Rock 2768* (GH); between Tengyueh and Lungling, *J.F. Rock 7174* (GH); no locality, *G. Forrest 18414* (GH); Ping-pien Hsien, *H.T. Tsai 61528* (GH); Shweli valley, *G. Forrest 8763* (E, GH). **INDIA.**East Bengal, *Griffith 3380* (A, GH, P); Jingale Bam near Nagahill, *Prain 769* (GH); Kachin Hills, Saden, Upper Burma, Mokim, *Shaik s.n*.; Ind. Or., *Griffith 286* (GH); Ceylon, Royal Botanic Gardens, Peradeniya, sect. C 276, *D.M.A. Jayaweera 1617* (GH). **INDONESIA.**Bali, Dedugul, *Dumat 414* (MO), Bali Timur, Tabana. 2 km W of Candi Kuning, in natural areas of Kebun Raya, beyond introduced Altingia forest, *McDonald & Ismail 4966* (E, GH); Dutch West Indies, *van de Koppel 3299* (MO); East Timor, Koepang, *De Voogd 1772* (A); Java, *Field Museum 373260* (A); Java, Ijoboshan, *C.S. Sargen s.n*.; W. Java, Nirmala Estate, gu Halimum area, Blukar and remnant of forest, *M.J. v. Balgooy 2912* (GH); West-Java, Res. Batavia. Pasir Tjarewed, Land Boland, west of Bogor (Buitenzorg), elev. 600 m., *Bakhuizen 6372* (MO); West Java, relict tree tp Tjibodas Mt. Garden, Gunung Gedeh (Mt.), *Willem Meijer s.n*. (MO); N. Sumatra, Karo plateau, Kaban Djahe, *J.A. Loerzing 17368* (A); South East Java, *H. O. Forbes 1201* (GH); Sumatra, Res. Benkaelen and Afd. Redjang, *T.H. Endert 1068* (A); Sumatra, Sumatra’s Westk. *Moera-Laboch For. Serv, Neth. Ind. 18066* (A). **MYANMAR.**East of Paungdaw Power Station, west bank of the Paungdaw chaung river,* J. Keenan 1407* (E), 1528 (MO); Patkai Mts., *G. Schaap 13* (A); gorge of the Hkrang Hka, North Triangle (Hkinhum), *F. Kingdon-Ward 20761* (A); south of Hpuginhku village, *J. Keenan 3679* (E); Tenasserim Division, Tavoy District, *J. Keenan 1940* (A). **THAILAND.** Nakhon Nayok, Khao Yai National Park, *T. Smitinand 10848* (E); NE Kjonkaen, Phu Khieo, Game Reserve, ca. 80 km E of Phetchabun, *Kyoto University 41655* (A).

#### 7. Liquidambar formosana

http://species-id.net/wiki/Liquidambar_formosana

Hance, Ann. Sc. Nat. Ser. V. v. 215, 1866. TYPE. CHINA: Taiwan: Martio 1864, R. Oldham s.n. (lectotype here designated, P [P00749063!]; isolectotypes: NY [NY00356137!], P [P00749064!]).

Liquidambar acerifolia Maxim., in Bull. Acad. Petersb. x. 486, 1866.
Liquidambar edentata Merrill, in Journ. Arnold Arb. viii. 6, 1927. (TYPE. CHINA: Fujian [Fukien]: probably near Foochow, *F.T. Metcalf & T.C. Chang 877*,fruit only(Lectotype here designated: UC [UC 288168!]).
Liquidambar maximowiczii Miq., Ann. Mus. Bot. Lugd. Bat. 3: 200, 1867.
Liquidambar rosthornii Diels, Bot. Jahrb. Syst. 29(3-4): 380, 1900.
Liquidambar tonkinensis A. Cheval. in Bull. Econ. Indochine, n. s. 10: 839, 1918.

##### Note.

A few specimens from Hunan and Guangdong exhibit pubescent stems, leaf surfaces and lobing attributable to young plants, previously annotated by Merrill (1934) as “*Liquidambar formosana* var. *heterolobata”*: *Tsang 20927* (MO, SYS), *B. Xiong 8845, 9492, 10032* (MO), Hupeh Prov.: *Wilson 513* (GH-2 sheets). This variety has never been formally published. These specimens are here referred to *Liquidambar formosana*.

The type of *Liquidambar edentata* Merrill is a mixture of a) *Liquidambar formosana* Hance [UC288168] as a single fruit mounted on the sheet, and b) a large branch of *Acer tutcheri* Duthie. Thus, lectotypification is required. One of the duplicates of *F.T. Metcalf and T.C. Chang 877* at UC [UC258487] bears a label with the original handwriting of Merrill: “*Liquidambar edentata* Merr.”, but the reference to the original publication (“Journ. Arnold Arbor. 8:6. 1927. Type in US Natl. Herb.”) is not Merrill’s own handwriting and was probably later added (as determined by John Boggan [US] from comparison with other holotype material at US described by Merrill). All duplicates of *Metcalf & Chang 877* [A 258487!, SYS 00072571!, UC 258487!] consist only of the branch of *Acer tutcheri*, without the *Liquidambar* fruit.

##### Distribution.

China (Anhui, Chongqing, Fujian, Gansu, Guangdong, Guangxi, Guizhou, Hainan, Hong Kong, Hunan, Hubei, Jiangsu, Jiangxi, Shanxi, Sichuan, Taiwan, and Zheijiang), Laos, and Vietnam. **Representative specimens examined**. **China: Anhui**: *K. Ping 1597* (MO); Chien Shan Hsien, Tien Chu Shan, Chien Shan Hsien, *C. S. Fan 280* (GH); Chiuhwashan, *S.C. Sun 1302* (GH); Wang Shilong. Hefei Shi, Zushan, east slope, *H.L. Yin 2031* (MO); Hwa Shan, *C.S. Fan 75* (E); **Chongqing** (formerly in Sichuan Prov.): Chengkou Shi, *T.L. Dai 103616* (MO); Nanchuan Hsien, *W.P. Fang 811* (A); **Fujian**: Fan Hsioh Niao, Shaowu and vicinity, *F.P. Metcalfe 9360* (GH); Hinghwa Dist., *H.H. Chung 1006* (GH); Nanping Shi, Mangdangshan, *G.-S. He 6179* (MO); Nanping Shi, 3800 Kan, *G.-S. He 5677* (MO); Minhow Hsien: Pehling, near village dwellings, *H.H. Chung 2132* (GH); Sing-Shan, Foochow and vicinity, *C.C. Tang 4655* (MO); Yenping: Buong Kang, *H.H. Chung 3589* (GH); Yenping: Cha-ping, on slope, *H.H. Chung 2901* (GH); **Gansu**: Wan Xian, Bikou, *Z.-Y. Zhang 14271* (MO); Wan Xian, Motianling Shan, Baishui Jiang Nature Reserve, ENE of city of Bikou, *D.E. Boufford et al. 37528* (MO);**Guangdong**: Chong Uen Shan near Kau Fung, Loh Ch’ang District, *W. Tsang 20927* (A, MO); Guangdong, Canton and vicinity, *C.O. Levine 1731* (MO); Lok F’au Mt., *C.O. Levine 1572* (MO); Lung T’au Shan, Iu village and Yeung uk village, *Lignan Team 12347* (MO); Nanling National Forest Park, *S. Ickert-Bond 1305* (F); Nanling National Forest Park, *S. Ickert-Bond 1305* (F), *S. Ickert-Bond 1309* (F); Nanxiong, *L. Deng 6602* (MO); Near Ninling city, on hill side, *S. Ickert-Bond 1321* (F); Road to Jiangxi, along river, ca. 5 km S of Shitang city, *S. Ickert-Bond 1326* (F); Road to Hunan from Nine Peaks, ca. 7 km outside of town, *S. Ickert-Bond 1324* (F); Wan Tong Shan, Ying Tak district, *T.M. Tsui 417* (MO); Yang Shan, and vicinity, South of Linchow, Yang Shan district, *T.M. Tsui 516* (MO); *T.M. Tsui 660* (2 sheets, MO); Xinyi Xian, *C. Wang 31824* (MO); Guangchow, White Cloud Hill on the way to the Temple, *H.H. Chung 866* (GH); **Guangxi**:* A.N. Steward 442* (GH);Chuen Yuen, *T.S. Tsoong 81972* (GH); Longjing, Daginshan, *P.X. Tan 57605* (MO); Loh Hoh Tsuen, Ling Yun Hsien, *A.N. Steward 29* (GH); Mts. Surrounding Pa Lau village, near Sui-Luk, SW o Nanning (Sui-luk village), *W.T. Tsang 21817* (GH); San-min village and vicinity, P’an-ku-shan and Ch’ao- t’ien-shan, Kwei-lin district, *W.T. Tsang 28068* (GH); Ta Tseh Tsuen, *A.N. Steward 1075* (GH);**Guizhou**: Jiangkou Xian, Baishuidong (white water cave) above the Minxiao River, SW of Jiangkou, *B. Bartholomew 773* (GH); Lungli, *H. Handel- Manzetti 185* (GH); **Hainan**: Bak Sa, *S. K. Lau 25962* (GH). Bawangshanling, *Z.-X. Li 3841* (MO); Chim Shan, Maan Ts’uen and vicinity, Ling Shui (Ling- tui) district, *H. Fung 20245* (MO); Ka Chik Shan, Ka Chik Shan and vicinity, *S.K. Lau 1645* (GH); Kam Kong, Yik Tsok Mau, *Canton Christian College Herbarium 7700* (MO); Lingshui Xian, Nanqui, *L. Deng 3116* (MO); Pak Shik Ling, Pak Shik Ling and vicinity, Ku Tung Village (Ching Mai district), *C.I. Lei 355* (GH); Yangxin Xian, Longgang Zhen, *C.-L. Ye 9689* (MO); Ya Xian, *X.-R. Liang 62343* (MO); Yonlin, Yaichow, *F.C. How 20124* (MO);**Hong Kong**: Chung Chi College, *S.Y. Hu & K.H. Yung 46* (MO); Chinese University of Hong Kong campus, *S.Y. Hu 20064* (MO); *S.Y. Hu 20953* (GH, MO); Hau T’ong Shan, Fuk Lung Monastery, Sin-Fung District, Fung Shue, *Y.M. Taam 779* (GH); Hong Kong University campus, *H.C. Tang 1473* (GH); Shing Mun Country Park, at the crossroads of Lead Mine Pass and Main Dam, *S. Ickert-Bond 1260* (F); **Hubei**: Changchow, White Cloud Hill on the way to the Temple, *H.H. Chung 866* (GH); Chikungshan, border of the provinces of Hupeh and Honan, on the divide between the Yang-Tze and the Hwai-ho rivers, *L.H. Bailey s.n*. (GH); Hinghwa Dist., *H.H. Chung 1006* (GH); Hupeh (W) Arnold Arboretum Expedition, *E.H. Wilson 795* (E); Lin District, *C.O. Levine 3302* (GH); *A. Henry 5218* (GH); *A. Henry 7630* (GH); Lung T’au Shan, Iu village and Yeung uk village, *Lignan 12347* (MO); Western Hupeh, Feng Heang, *E.H. Wilson 513* (GH); W. Hupeh, *E.H. Wilson 218* (GH); Xinyi Xian, *C. Wang 31824* (MO); Yang Shan and vicinity, South of Linchow, Yang Shan district, *T.M. Tsui 660* (MO); Yenping, Cha-ping, *H. H. Chung 2901* (GH); **Hunan**: Henyuang, Goulowshan, *J.B. Zuo 356* (MO); Liuyuang Xian, Longfa Zhen, Shizhu Feng, *B. Xiong 2922* (MO); P’ing T’ou Shan, T’ang Wan village, Yi Chang district, *W.T. Tsang 23613* (GH); Yushun Xian, Zhengxi, *X-G. Li 204950* (MO); **Jiangsu:** Changsu, *T.Y. Cheo 1149* (MO); Haichow village, *J. Hers 2264* (GH); Hua Shan, Nanking, *W.R. Carles s.n*. (E); Kinling, *E.H. Wilson 1639* (GH); Liu Liu Shan, near Haichow,* J*. *Hers 608* (GH); Mao Shan, Tanjang, *Tso 1803* (GH); Nanking, *S.S. Chien 1019* (GH); Yun-Tai-Shan, northern headland of Liuhe, extending into Kou Linhong, Lianyungong Bay,* SAYTBET 45208* (GH); Yun-Tai-Shan, Lian-yun-gang, NE of Jiangsu prov., *K. Yao 8505* (MO); Yuntai, Zikiang Shan (Purple Mountain), N extension of Mao Shan; NE of Sun Yat-Sen Memorial and Tomb, *SAYTBET 45272* (GH); **Jiangxi**: Chuen Yuen, *T.S. Tsoong 81972* (GH); De-Xin county, *K. Yao 11561* (GH); Gangmaiping Xiang Huangyangjie, *B. Xiong 5706* (MO); ); 3 km from Julianshan Nature Preserve entrance, *S. Ickert-Bond 1327* (F); Kinkiang, *E.H. Wilson 1628* (GH); Oo Chi Shan, near Lam Uk Village, Lungan district, *S.K. Lau 4809* (GH); San-min village and vicinity, P’an-ku-shan and Ch’ao-t’ien-shan, Kwei-lin district, *W.T. Tsang 28068* (GH); Sang-su-ling, near Sih-cha-chieh Kan River, about 60 mi south of Nanchnag, Kinagsi, *H.H. Chung 40* (GH); **Shanxi**: Yuyang Xian, Xiaoguojiaba, *K.-J. Fu 5799* (MO); **Sichuan**: *T. T. Yu 229* (GH, MO); **Taiwan**: Chiayi Hsien, Fanlu Hsiang, area nearby Pantienyen, *Y.-R. Lin 516* (MO); Formosana Hokuto, *A. Faurie 279* (GH); Hsinchu Hsien, Wufengm Wushishan, *S. Saito 8371* (MO); Kelung, *O. Warburg 9810* (GH); Nanto, Province Nanto, *E.H. Wilson 10031* (GH)Wuu Tsau street, *T. Sozan 13490* (GH), *L.L. Liu et al. s.n*. (MO); Taipei, University campus, *Y.R. Cheun s.n*. (GH, MO); Tamsui, *A. Henry 425* (GH); Taitum, *U. Faurie 45* (GH); **Zheijiang**: Feng Yang Mountains, *H.-Y. Zou 140* (GH); Kwangsi, Yung Hsien, Ta Tseh Tsuen, *A.N. Steward 1075* (GH); Langquan, Taishui, *R.C. Ching 4823* (GH); Lishui, Dagantou, *S. Chang 6242* (MO); Sang-su-ling, near Sih-cha-chieh Kan River, about 60 li souht of Nanchnag, Kinagsi, *H.H. Chung 40* (GH); Taichow, *R.-C. Ching 1578* (GH), *R.-C. Ching 4823* (GH); Tien Tai Shan, Kwohchingze, *C.Y. Chiao 14238* (GH). **LAOS:** Bolikhamsay, Khamheut district, Ban Namphao, ca. 5 km east of town proper, *D.D. Soejarto 11399* (GH); haut plateau, bassin d’ attopen, *Harmand 13007* (P). **Vietnam:** **Cao Bang**: Ha Lang, municipality Thang Loi, vicinity of Thang Loi village, *P.K. Loc et al. 1704* (MO); **De Quang**: Quang Tri, Lao Bao, *M. Poilane 1317* (P);**Bac Giang**: Sau (Annam), Tonkin, foret de Pho-ve, *A. Chevalier 2964* (P); **Ha Tay:** Da Chong, *A. Petelot 5747* (A); Mt. Bavi National Park, close to Park headquarters, roadside, *S. Ickert-Bond 1290* (F), Mt. Bavi National Park, roadside, *S. Ickert-Bond 1291* (F); **Hoa Binh**: Kim Boi, *T. Tien Phuong 2539* (HN); **Lang Son:** Dong Dang, *B. Balansa 1156* (P); Huu Lien District, Huu Lsien Municipality, Huu Lien Protected Area, near village of Lan Cau, *D.K. Harder et al. 4180* (MO); Lai moi sau cuoi, Savanne cay go, Chi lang, *N. Tang Khoi 420, 421* (HN); **Nghe An**: Ke hhe, *Donnat 38180* (P); Reserve forestier de Co - Ba (Vie-Nhe), *F. Fleury 30170* (P); **Ninh Binh**: Cuc Phuong National Park, headquarters, east of helipad, *N.M. Cuong 93* (GH, MO); **Phu Tho**: Foret de Dao gia, pres de Phu Tho, *A. Chevalier 37471* (P); **Than Hoa**: Phong Y’, *M. Poilane 1610* (P); **Tuyen Quang**: Reserve forestiere de Niu-La, *F. Fleury 37961* (F); **Vinh Phuc**: Ngoc Thanh, Me Linh, *Phuong 4647* (2 sheets, HN).

#### 8. Liquidambar gracilipes

(Hemsl.) Ickert-Bond & J. Wen comb. nov.

urn:lsid:ipni.org:names:77134709-1

http://species-id.net/wiki/Liquidambar_gracilipes

Basionym: *Altingia gracilipes* Hemsl., in Hook. Ic. Pl. t. 2837, 1907. TYPE. CHINA: Fujian, *S.T. Dunn* Hb. Hongk. 2682. (Lectotype here designated HK [HK 10948!]; isolectotypes: A [A 00043390!], IBSC [IBSC 001001!])

Altingi gracilipes Hemsl. var. *serrulata* Tutcher, Hong Kong Administrative Report 1914: M31. TYPE. CHINA, Hong Kong, Ukautang, *W.J. Tutcher* Herb. Hongkong No. 10947) (holotype: HK [HK 10949!, collected 25/5/1914]; isotypes: HK [Garden Department Hong Kong 1711!, collected 25/5/1914], HK [HK10950!])
Altinga gracilipes Hemsl. f. *uniflora* H. T. Chang, Sunyatsenia 7: 74: 1948. TYPE. CHINA, Fujian, *Y. Ling 2295* (holotype: IBSC 001003!)

##### Distribution.

China (Fujian, Guangdong, Hong Kong, and Zhejiang). **Representative specimens examined.** **CHINA:Fujian**: Jian Ou, Wang Mu Lin, *Fujian Forestry Coll. 41* (MO); Long Yan, Jiang Shan, Shuang Che, *H. Chen 1272* (F, MO); Nan Ping, Mang Tang Mountains, *G. He 4690* (MO),* G. He 5479* (MO),* G. He 6155* (MO); Yong An, Hong Tian, *Fujian Team 5567* (MO); Yungyung Mts., Fujian, central, *S.T. Dunn 2682* (GH); **Guangdong**: Chaochow district, *N.K. Chun 42718* (MO); Da Pu, Yan Shang, Min Ying, *L. Deng 5438* (MO); Da Pu, Feng Xi,* X. Wang 153* (MO); Da Pu, Ying Jian, *Z. Li 963* (MO); Da Pu, Pu Cheng, *X. Li 202545* (MO); Feng Xun, Da Tian, Bei Xi, *X. Li 200921* (MO); He Yuan, Xin Feng Jian, *B. Yu 103106* (MO); He Ping, Qing Zhou, *Z. Wei 120579* (MO); Jiu Lian Xiang, Xiao Shui, *Z. Wei 120217* (MO); Lian Ping, Zhong xin, Da Shui, *Y. Liu 242* (MO); Ping Yuan, *L. Dong 4331* (MO); Qiao Lin, Shi Hu, *L. Dong 4685* (MO); Raoping Xian, *N.K. Chun 42718* (2 sheets, MO); Rao Ping, Feng Huang, *X. Li 200701* (MO); Rao Ping, Feng Huang Yu, *H. Zeng 72101* (MO); Tung Koo Shan, Tapu district, Tan Shue, *W.T. Tsang 21697* (GH, HK, P); Wu Hua, Shuang Hua, *Z. Li 1162* (MO); Wu Hua, Qi Mu Zhang, *Y.-J. Wang 56484* (MO); Xin Yi, Da Wu Lin, *Z. Feng 36293* (MO); Yam Na Shan [Yit Nga Shan] Mei [Kaying] District, *W.T. Tsang 21514* (GH), Yunfu Xian, *C. Wang 37057* (MO); **Hong Kong**: *M.T. Sin & W.K. Woo s.n*. (HK); Pat Sin Leng Country Park, Nam Chung trail, *S. Ickert-Bond 1272* (F); Nam Chung, *D. Lau 43* (GH, MO), *T.W. Lau 98* (GH, MO), *Y.-W. Lam 1429* (HK), *K.-L. Yip 4082* (MO); Pat Sin Leng, Plover Cove Country Park, Plover Cove Reservoir, *S. Ickert-Bond 1266* (F); Pat Sing Leng Nature Trails, *L.T. Lo 629* (HK); Sha Tau Kok-luk Keng, *S.Y. Hu 9984* (GH); Wu Kau Tan N.T., *S.Y. Hu & Y.C. Kong 175* (MO), *Y.S. Lau 2634* (HK); **Zhejiang**: Between Ping Yung and Tai Suan, *R.-C. Ching 2199* (E); Feng Yang Mtn., *H.-Y. Zou 79* (MO),* H.-Y. Zou 151* (GH), *H.-Y. Zou 762* (GH); Long Qian, Ju Shui, *P.L. Chiu 1072* (MO); Qing Yuan, Wu Du Mountain, *Z.G. Mao 10231* (MO); Shui Chang, Pu Nang, *P.L. Chiu 1470* (MO); Taishun Hsien, *Y.L. Keng 316* (GH).

#### 9. Liquidambar multinervis

(Cheng) Ickert-Bond & J. Wen comb. nov.

urn:lsid:ipni.org:names:77134710-1

http://species-id.net/wiki/Liquidambar_multinervis

Basionym: *Altingia multinervis* Cheng, in Notes For. Inst. Nat. Centr. Univ. Nanking, Dendrol. Ser., No. 1, 3 1947. TYPE. CHINA: Guizhou: Chishui, Tiantaishan, 29 Oct 1938, *P.C. Tsoong 256* (holotype, N seen as photo!; isotype, SYS 72729!).

##### Note.

Specimens of *Liquidambar multinervis* show long-petioled papery leaves, with 10 lateral veins, and serrate margin, closely resembling *Liquidambar siamensis* or *Liquidambar excelsa*. Broken infructescences of the specimen at N suggest few fruits per infructescence, thus underscoring the closeness to *Liquidambar siamensis*, while Cheng (1947) described its close affinity with *Liquidambar yunnanensis*. This species is poorly understood, we have only seen the type collection that consists of a branch with multiple leaves and a crushed infructescence. The distributional discontinuity of *Liquidambar multinervis* in N Guizhou from those of *Liquidambar siamensis* and *Liquidambar excelsa* much further south may warrant specific status, but more material is needed. N Guizhou is not well explored botanically and with more exploration of this area, we might be able to better characterize this taxon in the future.

##### Distribution.

China, N Guizhou.

#### 10. Liquidambar obovata

(Merrill & Chun) Ickert-Bond & J. Wen comb. nov.

urn:lsid:ipni.org:names:77134711-1

http://species-id.net/wiki/Liquidambar_obovata

Basionym: *Altingia obovata* Merrill & Chun, in Sunyatsenia, 2: 238, 1935. TYPE. CHINA: Hainan: Yaichow, Ngai Yen, *F.C. How 70369* (holotype: NY [NY 00356124!]; isotypes: A [A00043391!], HK [HK1713!], SYS [SYS 00095710!]).

##### Distribution.

China, Hainan. **Representative specimens examined**. **CHINA:Hainan**: no locality, *H.Y. Liang 64371* (E), *H.Y. Liang 64734* (GH), *H.Y. Liang 62594* (P); *C. Wang 35691* (MO); *C. Wang 35897* (GH); Ding’an Xian, *C. Wang 36153* (MO); Lingshui Xian, *C. Wang 36638* (MO); Mo San Leng, *N.K. Chun 44321* (GH); Qiong Zhong county, Cheng Po district, Da Li village (up?), Baishui Ling, Deng, *Liang 3685* (MO); Waning County, Liulian Mts., *Y. Zhong 4321* (MO); Waning county, Wumie district, Tongtie mountain (Ling), *Z. Li 4972* (MO).

#### 11. Liquidambar orientalis

Mill.

http://species-id.net/wiki/Liquidambar_orientalis

Gard. Dict. ed. 8, n. 2, 1768.

Liquidambar imberbis Ait., Hortus Kew. (W. Aiton) 3: 365, 1789.
Liquidambar orientalis Mill. var. *integriloba* Fiori, Ann. R. Inst. Sup. Agr. For. Naz. 9: 153, 1924.

##### Note.

*Liquidambar orientalis* still needs lectotypification. The prologue states as follows: *‘The seeds were sent by Mr. Peyssonel from the Levant, to the French king’s garden at Marli, a few of which were sent to me by Mr. Richard, the king’s gardener, which succeeded in the Chelsea [Physic] garden.’*

When examining type material from FI of var.* integriloba* and the typical *Liquidambar orientalis* no clear distinction of lobing was observed. The FI specimens were identified as *Liquidambar orientalis* var. *integriloba* (*A. Fiori 230* – 2 sheets, *A. Fiori 231* – 2 sheets, G. Jannone s.n. – 1 sheet, *A. Fiori* s.n. – 1 sheet). When comparing material from Turkey (ISTO), the specimens collected by *Aksoy 5202* (2 sheets) have margins that are sometime lobed beyond the typical 5 –lobing, also observed in *Aksoy 5201* (ISTO-1 sheet), and *Aksoy 5203* (ISTO - 4 sheets), while specimens identified as *Liquidambar orientalis* var. *integriloba* lack such lobing, as seen in *Aksoy 5204* (ISTO-3 sheets) also from Turkey. This specimen (*Murray 1020* – GH, MO) lacks the typical lobing of var. *orientalis* and could thus be considered var. *integrifolia*.

##### Distribution.

Southwestern Turkey and on the Greek island of Rhodes. **Representative specimens examined.** **Turkey:** C_2_ Mugla: Kaycepra, *Goner 9145* (MO); C_2_ Mugla: Kargi, 10 km N of Fethiye, *G. Polunin 14923* (E); Mughla near Dogusbelen, *Danish Bot. Trans-Asia Expedition III, No. 2081* (E); Mugla, between Köycefiz and Kavak *Aksoy 5203 (ISTO)*; Mugla: Distr. Marmaris, Er. Koezcegiz, *Khan 45* (E); Isparta, Sütçüler, Karacaören *Aksoy 5204 (ISTO)*; Koezcegiz, *J.S. Andersen 2081* (E); near Severagno, *Khan et al. 45* (E); Paludal place (marsh), 1 km NE of Marmaris, *E. Murray 1020* (A, E, MO); Vil. Mughla near Dogus belen, *P.H. Davis 13474* (E).**GREECE:** collini a sud di Severagno, *G. Jannone s.n*. (FI); Peveragno, secus rivulum “Pelicano”, *K.H. Rechinger 8550* (E); convento d’Iskiati, *A. Fiori 130* (FI), *A. Fiori 230* (FI); era Alaeruna ed Apallaua, lengo il fiemme Saduras, *A. Fiori 231* (FI); Rhodes Island, between Malona and Archangelos, *K. Boratynska et al. 15* (K); between Malona and Archangelos, old trees along small stream, very frequent, *K. Boratynska 164* (K); Salakos, hedges near stream, *Davis 40317* (K); SE of Salakos, along stream, below orchard, *K. Boratynska 15* (K). *Cultivated*: Italy: Rome, *Martinetto* s.n. (ASU). USA: Washington, University of Washington Arboretum, *A.L. Bogle 1561* (ASU).

#### 12. Liquidambar poilanei

(Tardieu) Ickert-Bond & J. Wen comb. nov.

urn:lsid:ipni.org:names:77134712-1

http://species-id.net/wiki/Liquidambar_poilanei

Basionym: *Altingia poilanei* Tardieu, Fl. Camb., Laos & Vietn., Fasc. 4, 95 (1965), in adnot. TYPE. VIETNAM: SaPa, *M. Poilane 12844* (holotype, P 00317366!).

##### Distribution.

Vietnam. **Representative specimens examined**: **VIETNAM. Lao Cai**: Sa Pa, *China-Vietnam Team 8462* (HN); Ta Pinh Hmong village, some of the last remaining forest by small river across from rice paddies, *S. Ickert-Bond 1296* (F).

#### 13. Liquidambar siamensis

(Craib) Ickert-Bond & J. Wen comb. nov.

urn:lsid:ipni.org:names:77134713-1

http://species-id.net/wiki/Liquidambar_siamensis

Basionym: *Altingia siamensis* Craib, in Kew Bull. 1928, 68. TYPE. THAILAND: Mŭ ang Pan, 700 m, evergreen forest, Doi Duan, *Kerr 5110* (holotype: BK257949!).

Altingia tenuifolia Chun ex H.T. Chang, in Acta Sci. Nat. Univ. Sunyatseni, 1959(2): 34, 1959. TYPE. CHINA: Guizhou [Kweichow], Dushan, *Y. Tsiang 6677* (holotype, IBSC!, isotypes: A [A00043392!], E [E00181734!], NY [00356122!]).
Altingia angustifolia H.T. Chang, Acta Sci. Nat. Univ. Sunyatseni, 1961 (4): 52, 1961. TYPE. CHINA: Guangdong, Dapu, in dense forests, 10 Jun 1957, *L. Deng 5031* (holotype: IBSC (IBSC 001000!); isotype: PE (PE00029853!).
Altingia takhtajanii Thai, Bot. J., URSS, l. 996, 1965. TYPE. VIETNAM: Chieng-ve, Moc-chan, alt. 770 m, 13-III-1963, *Thai Van Trung 108* (holotype: LE!)

##### Distribution.

China (Guangdong, Yunnan), Cambodia, Laos, N Thailand, and Vietnam. **Representative specimens examined. CAMBODIA.**Forét de Phnom Penh, Komnhan, *M. Bejaud 877* (P). **Kampot**: Bokor National Park, Pokopvil waterfall, near the head, *S. Ickert-Bond 1280* (F),* 1281* (F); Kampot, Bokor National Park, upper Popovill waterfall head, *M. Monyrak 10* (A); **INDONESIA.**Java, Preanger Takoka, *Koordes 15754* (P). **LAOS:** Fam Neva et M. Ham, *M. Poilane 2000* (A, P); haut cours de la Zehepone entre A Chieng et Klem Zalo, *M. Poilane 13500* (P); Pak Song, Sedone Prov., Sedone, *J.E. Vidal 4461* (P). **THAILAND**. **Nakorn Nayo:** Khao Yai, *Hardial 601* (A), *J.F. Maxwell s.n*. (MO); Nakhon Nayok, Khao Yai National Park, *T. Smitinand 10848* (E); Nam Phnom, Prov. E., District Chaiyaphum, *C.F. van Beusekom 4102* (MO). **Union of Myanmar**:Tenasserim division, Tavoy district, east of Paungdaw Power Station, west bank of the Paungdae chaung, *J. Keenan 1407* (E, MO). **VIETNAM:**semi flumen Da one in foret Bieu Loa, *L. Pierre s.n*. (P); **Da Nang:** Tourane, 100 km S of Hue, the later being Loureiro’s type locality for the majority of the Cochinchina species, *J. Clemens 3388* (A, MO, P); **Gialai-Kontum**: Dac Long, Dac Glai, Kontum, *N. Kin Dao 182* (HN); Kbang, Kong Ha Nung, So Nglang, Xa Dong, Dac Glay, va mot so tinh Khac, *no collector 745* (HN); So Rang, An Khe, *V. Xuan Phuong 586* (HN); **Khánh Hòa:** Cay to hop, Nhatrang, *M. Poilane 3228* (P); Hon Ba Mtns., Suoi Cat Village, *D.D. Soejarto DDS 13561* (MO); Nui Chua National Park, Ninh Hai Distr., Vinh Hai Municipality, *J.C. Regalado et al. HLF4449* (MO); **Lam Dong:** en peu au sud de la Mation agu cole de Blao pres du Haut Donai, *M. Poilane 22153* (P); Loc Tan, Bao Lac, *N. Tien Ban 469* (HN), *H. Tue 523* (HN). **Ninh Thuan**: Ka Rom pro: Phanrang, *M. Poilane 9938* (2 sheets, P); **Son La:** Song Ma, *N. Tien Ban 110* (HN).

#### 14. Liquidambar styraciflua

http://species-id.net/wiki/Liquidambar_styraciflua

L., Sp. Pl. 999, 1753. TYPE: *Kalm*, Herb. Linn. No. 1134.1 (LINN), (lectotype designated by Wijnands in Bot. Commelins: 109. 1983, [LINN HL1134.1, seen as image!]).

Liquidambar barbata Stokes, Bot. Mat. Med. iv. 332, 1812.
Liquidambar gummifera Salisb., Prod. 393, 1796.
Liquidambar macrophylla Oerst., Am. Centr. 16 t. 10, 11, 1863. TYPE. NICARAGUA: Monte Pantasmo. *A.S. Oersted 3050* (lectotype designated by Sosa in Flora Veracruz 1:2. 1978 [C], as photo at MEXU; isolectotype [F!]).
Liquidambar styraciflua var. *macrophylla* (Oerst.) Nied. in Engl. & Prantl, Nat. Pflanzenfam. 3, Abt. 2a: 124, fig. 69H, 1891.
Liquidambar styraciflua var. *mexicana* Oerst., Amer. Centr. 16, t. 11. 1863. TYPE. MEXICO: Veracruz: H. Bartholomé, *F.M. Liebmann 3052* (lectotype designated by Sosa in Flora Veracruz 1:2. 1978 [C], as photo at ALA! and XAL).

##### Representative specimens examined.

**Belize**: **Cayo**: Chiquibul, Ceibo Grande to Main Divide, *A.K. Monro 2626* (MO), Ceibo Grande to drill sites track, *M. Pena 1046* (MO); Chalate, Vicinity of La Palma, Dept. of Chalatenango, *P.H. Allen 7265* (F). **El Salvador:** **Dept. Chalatenango**: Along trails from San Ignacio to Las Pilas, west slope, *J.M. Tucker 1209* (F); Chalate, El Jute, *S. Calderon 1928* (F); Hacienda San Miguel near Metapan, *M.C. Carlson 757*, (F); On the road between San Salvador and La Palma, *M.C. Carlson 608* (F); Salvador, Ana, forested slopes between Rio San Miguel and summit of Cerro El Pinal, Pine-aok zone, mountains near Finca San Jose, 10 km, *N.C. Fassett 28302*, (F, GH, MO); San Ignacio, La Palma, *M. Hernandez 558* (MO); San Jose Igenio, P.N. Montecristo, *V.M. Martinez 142, 454*, (MO); 20 kms al SO de Montecristo, *R.A. Molina 12578* (F). **Guatemala:** Cerro Negro, *J.A. Steyermark 51726* (A); Chicoyonito, Dept. Alta Verapaz, *J.D. Smith 1855* (GH); Cocola, Reion de Cocola, northeast of Carcha, Dept. Alta, *P.C. Standley 70309* (A); Dept. Alta Verapaz, Coban, *H. Tuerckheim 1804* (E, GH, MO); Dept. Huehuetenango: Cerro Victoria, *J.A. Steyermark 49713*, (A); Dept. Zacapa: pine-covered slopes, Sierra de las Minas, *J.A. Steyermark 29732* (A); Dept. Zacapa: along Rillito del Volcan de Monos, *J.A. Steyermark 42409*, (A); Huehne, Yalambohoch, *E. Seler 3024* (GH); Nebaj, Dept. Quiche,* A.F. Skutch 1739* (A); Sierra de las Minas, near San Geronimo, *W.A. Kellerman 6412* (MO); Valley of Rio de las Violetas, north of Nebaj, *G.R. Proctor 25212* (F, MO); Verapaz, Baja Verapaz. Mun. Chilasco, 6km al SW de Chilasco, *L.P. Tenorio 14900* (MO). **Honduras:** Aldea El Carmelo, 1 km SE of Valle de Angeles, *G. Amador 172* (F); Floresta de pino-liquidambar de la Monatana Zanquin, *R.A. Molina 2834* (F, GH); Lempira, Celaque National Park, ca. 7 km of Gracias, *T.F. Daniel 9628* (MO); Rio Pijol Valley, 6-7 km south of Nueva Eperanza, *R.L. Liesner 26609* (MO); Trencheras, 20 km N of Siguatapeque, *R. Howard 627* (A); Valle Encantado, slopes of Mt. Uyuca, *P.H. Allen 11181* (A); **Dept. Morazan**: entre Pena Blany, Lo de Ponce, *L.O. Williams 17113* (GH); slopes of Cerro de Uyuca, *P.C. Standley 887* (F); **Dept. Altantida**: vicinty of La Ceiba, near Danto river, *T.G. Yuncker 8776* (GH); **Dept. of Comayagua**: edge of ravine near El Achote, *T.G. Yuncker 5830* (F, GH); El Achote, near Siguatepeque, *P.C. Standley 56158* (A); **Comaya**: bosque mixto y humedo de Barranco Trincheras, *R.A. Molina 10807* (F); R. Selan, *V. J. Rodriguez 2840* (F); vicinity of Siquatepeque, *P.C. Standley 6497* (F); **Cortes**: Montana de Cusuco, Cordillera de Idalfonso, *R.A. Molina 7254* (F); Montaña San Cristobal, sur de Agua Fria, *R.A. Molina 7614* (F); Montaña Agua Fria, *R.A. Molina 11342* (F); Montana San Idalfonso entre Banaderos y Cusuco, *R.A. Molina 11454* (F). **El Paraiso**: Guinope, *V.J. Rodriguez 1883* (F); Montaña Teupasenti, entre Junquillo y Teupasenti, *R.A. Molina 11940*, (F); Mt. Volcan, *L. Williams 12190* (F); Paraiso; **Lempira:** Montana de Celaque, SE portion of massif, *G. Davidse 34570* (MO); Mount Elaque National Park, *J. Renfrow 16* (MO); **Moraza**: 20 km de Tegucigalpa, Montanña La Tigra, *A. Rubio 63* (MO); Bosques del Volcan de Guaimaca, Cordillera Misoco, *R.A. Molina 6127* (F); Campamento de Las Flores, *P.C. Standley 13708* (F); Cerca de Montaña La Tigra, *R.A. Molina 13755* (F); Faldas de Uyuca, *R.A. Molina 983* (F); Los Planes. 25 km al N.E. de Tegucigalpa, *S. Y. Chevez 119* (MO); Pinares entre La Piramide y Zambrano, *R.A. Molina 11034* (F); Region of El Quebracho, *P.C. Standley 23747* (F); Valle Encantado, slopes of Mt. Uyuca, *P.H. Allen 11181* (F); **Ocotep**: Cordillera Merendon 10 km from Nueva Ocotepeque, *R.A. Molina* 22235 (F**)**; **Olanch**: Montaña La Bellota en Cordillera Almendares, *R.A. Molina 8430* (F); **Siguate**: 7.5 km SW of Gracias, Lempira. Celaque National Park, *T. Hawkins 176* (MO); Alrededor del Centro de Visitantes, *D. Mejia 357* (MO); Guamil alrededor de Los Planes, *D. Mejia 402* (MO). **Mexico: Chiapas**: A 500 m al N de Rayon, camino de Pichucalco, *S.E. Martinez 24118* (MO); 3 km northwest of Pueblo Nuevo Solistahuacan, *R.F. Thorne 40034* (MO); at Rincon Chamula, 12 km northwest of Pueblo Nuevo, *P.H. Raven 19784* (F); Colegio Linda Vista (Yerba Buena), *G.L. Webster 17747* (MO); Colonia Kokijaz, *A. Mendez Ton 6087* (MO); Mpio. De Bochil, Puliupul, *C.H. Perino 3262* (MO); Ridge with Montane Rain Forest- Pine Oak, *D.E. Breedlove 21762* (MO); San Andres Larrainzar,* L.G. Gonzalez 233* (MO); Steep slope with montane rain forest, *D.E. Breedlove 34365* (MO); West of Tenejapa Center, *D.E. Breedlove 6886* (F); **Hidalgo**: 5 kms al oeste de Tianguistengo, *M. Hernandez 5630* (MO), *M. Hernandez 6912* (MO); 5 kms al oeste de Tianguistengo, district Zacualtipan, *H.E. Moore 1925* (GH); 4 kms al oeste de Tianguistengo, *(styraciflua)* (MO); along Highway 85, *D.H. Norris, 17397* (MO); district Jacala, municipality Chapulhuacan, *H.E. Moore 2176* (GH); district Molango, municipality Molango, *H.E. Moore 1995* (GH); **Nuevo Leon**: Dulces Nombres, *F.G. Meyer 2799* (MO); **Oaxaca**: 12 km al N de Guevea de Humboldt, distr. De Juchitan, *R. Torres 2541* (F); Distrito Mixe: Municipio de Totontepec: Totontepec, *Reyes Rivera, J. 257* (MO); Loma del Guayabo, Huautla de Jimenez, *M. Hernandez 448* (MO); Municipio de Santiago Comaltepec: La Esperanza, *R. Lopez Luna 27* (MO); Municipio de Totontepec: Totontepec, *R.J. Rivera 907* (MO); Municipio de Totontepec, Chinantequilla, *E. Vargaz Ruiz 44, 124* (MO); **Puebla**: Region Orizaba (second label states Xalapa), *M. Bourgeau 2412* (GH); **Tamaulipas**: Rancho del Cielo (property of Frank Harrison), ca. 7 km WNW of Gomez Farias, *W. Burger 26* (F); En el Rancho El Julilo, *S.E. Martinez 3872* (F); **Veracruz**: 1 km above and NW of San Andres Tlalnehuayocan, *M. Nee 26198* (F); 1 km al norte de Banderilla, Mun. Banderilla, *J.I. Calzada 5246* (F); 1 km NW of Elotepec along (impassable), *M. Nee 28898* (F); 2 km al NE de Banderilla, Rancho La Mesa, Banderilla, *M.G. Zola 500* (F, MO); 3 km SSW of Zongolica along gravel road to Chichiquila, Mun. Huatusco, *M. Nee 29442, 29444* (F); 6 km (by road) ESE of Ixhuacan de Los Reyes, Mun. Ixhuacan de Los Reyes, *M. Nee 22484* (F, GH); 10 km north of Huatusco in typical *Liquidambar* forest , *G.K. Arp 4186* (F); 18 mi North of Jacala on Highway 85, *D. Seigler 3601* (F); along Huayacocotla--Zontecomatlan rd., between Barro, La Calabaza and 5 km by road SE of Zilacatipan, Mun. Huayacocotla, *M. Nee 26885* (F); along very winding road from Naolinco to Misantla, 13 km by road S of turnoff to Yecuatla and 6 km by road N of Paz de Enriquez, Mun. Yecuatla, *M. Nee 26393* (F); about 8 mi N of Teziutlan, toward El Mohon (near border of state of Puebla), *E.W. Manning 53823* (GH); between Coscomatepec and Huatusco, *F. Boutin 3480* (F); Camino Bastonal a Santa Marta, *Gomez-Pompa 5382* (F); Camino a Rancho Nuevo, Huayacotla, *M. Hernandez M 1499* (F); Cerca de San Fernando, camino a San Fernando, *O.R. Ortega 1234* (F); Cerro de Macuiltepetl, Xalapa, *M.G. Zola 697* (F); Cerro de San Martin, *J.I. Calzada 539* (F); Cerro de Villa Rica cerca de Mundo Nuevo, *C.G. Castillo* 1803, (F); Coacoatzintla, *R. Ortega 837* (F); Consolapan, 4 km al norte de la desviacion, *J.I. Calzada 5250* (F); en el volcan Santa Marta a 25 km al N de Catemaco, *S.E. Martinez 3972* (MO); entre Zongolica y Nepopoalco, Zongolica, *T.V. Vazquez* 153 (F); gorge at Puente Acabaloya, ca. 1 km SE of Xico Viejo, *M. Nee 26295* (F); ); Jalapa, *C.G. Pringle 7754* (GH), *C.L. Smith 1778* (GH); Jardin Botanico y Arboretum de Insitituto de Investigaciones Sobre Recursos Bioticos (INIREB), ca., 4km SSW of Xalapa, *H.H. Iltis 942* (F); kilometro 7, carretera San Adresito, Xalapa, *M.G. Zola 616* (F); Lado SE de Laguna Catemaco, *J.H. Beaman 5148* (F); Los Tuxtlas, Ocotal Grande, 5 km N de Mecayapan, Ibarra,* G. Manriquez 2339* (MO); Mun. Juchique Ferrer La Cima, Plan de las Hayas, *M. Hernandez 1610* (F); Mun. San Andres Tuxtla, Cerro Vigia al E de Volcan San Martin, *J.H. Beaman 6276* (F); Mun. San Andres Tuxtla, cima del Volcan San Martin, *J.H. Beaman 5970* (F); Mun. Xalapa, Jardin Botanico Clavijero, 3 km SE of Xalapa, *M. Nee 29700* (F); Nacaxtla, Zongolica, *T.V. Vazquez 260* (F); N Banderilla, *J. Dorantes* (MO); Bastonal-Sierra Santa Marta road, *A. Gentry 32417* (MO); Near Highway 130 between Huachinango and Tulancingo, *J. Conrad 3262* (F); Near Huatusco, *F. Boutin 3494* (F); Nogales, *E. Matuda 1157* (MO); Parque Ecologico of the Jardin Botanico Fco. Javier, Clavijero, 2km SW of Jalapa along road to Coatepec, *M. Nee 23457* (F); Paz de Enriquez cloud forest, 15 km south of Misantla, *L. Bohs 1798* (GH); Orizaba, *Botteri 860* (GH); Paxopec, Municipio de Cacoatzintla, *F.A. Ventura 19250* (MO); Rancho del Mesa, Municipio Banderilla, *R.W. Marquez 997* (F); Rancho Nuevo entre Plan de las Hayas y Tierra, *C.G. Castillo 1350* (F); Ridge on S side of gorge of Cascada de Texolo, 3 km SE of Villa Xico (Xico=Jico), *M. Nee 26005* (F); Road to microwave station from Rancho Chula Vista near Coscomatepec, *F. Boutin 3485* (F); Sierra de Sta. Marta, *M. Sousa 3548* (F, MO); Loc. Choapan, Mpio. Santiago Choapan, *L.P. Tenorio 5329* (MO); Tenejapa, carretera Huatusco - Coscomatepec, *R. Avendaño 277* (F); Tlalneuayocan, *M.G. Zola 651* (F); Vaxin, cerca del Volcan San Martin, *M. Sousa 3433* (F, MO); vicinity of small dam, 1/2 km W of Cinco Palos and 8 km NW of Consolapan, Mun. Jalapa, *M. Nee 29689* (F); Xonamanca, Zongolica, *T.V. Vazquez 286* (F); Camino al Sumidero, Xalapa, *M.G. Zola 746* (F). **Nicagaragua: Jinotego**: Esteli, N slope of Cerro El Fraile, *W.D. Stevens 18095* (MO); Finca Aventina, in sierra east of Jinotega,* P.C. Standley 10026* (F); Hacienda La Balestina, situada a unos 10 km E de la ciudad de Jinotega, *A. Grijalva 250* (MO); Macizos de Penas Blancas, *W.D. Stevens 11510* (MO); N slope of Volcan Yali, *W.D. Stevens 15074* (MO; **Madriz**: 5 km SW of San Juan de Rio Coco, *W.D. Stevens 17665* (MO); 5.2 km N of San Fernando, *W.D. Stevens* (MO); Cerro El Fraile, *P.P. Moreno 22766* (MO); **Matagalpa**: 5 km east of Yucul, *C.E. Hughes 330* (MO); Cerca de entrada a bosque de Selva Negra, *M. Araquistain 3538* (MO); El Ocotal km 134 between Matagalpa and Santa Maria, *R.A. Molina 20435* (F, MO); Macizos de Penas, Blancas, SE side, *W.D. Stevens 21054* (MO); Sta. Maria de Ostuma, Cordillera Central de Nicaragua, *L.O. Williams 28004*, *23388* (F); **Wiwili**: Deparmento de Nueva Segovia: ca. 5.2 km N of San Fernando, valley of Rio San Fernando, *G.W. Stevens 3248* (MO); Jinotega, Municipio de Wiwili, Reserva Natural Kilambe, Communidad Aguas Frias, sector Caballo, *R. Rueda 16311* (MO); **Nueva Segovia**: Cerro Mogoton, *J.T. Atwood 9* (MO), *D.A. Neil s.n*. (F); Cerro Mogoton, 0.5 km W of El Volcan, 3.5 km NE of Dipilto, *S. Tomlin 176* (MO); Dipilto, El Placer, km 247 carretera a Las Manos, *P.P. Moreno 25910* (MO); Rio Arenal de Yali, Jalapa, *J.T. Atwood 6816* (MO). **U.S.A.: Alabama**: Alabama Biological Survey, Auburn, Earle, F. S., (MO); Alabama, Rt. 11, near Knoxville, *C.F. Reed 102737* (MO); Bienville Blvd., near Cadillac Sq., Dauphin Island, *R. Deramus 1043* (MO); Rt. 21, 1 mi S of Riedmont, *C.F. Reed 149022* (MO); Rt. 31, near Stapleton, *C.F. Reed 103825* (MO); **Arkansas**: 0.8 mi north of Arkansas-Louisiana state line; Hulton, *B.F. Bush 2425* (MO); Boston Mountains, *P.H. Raven 26323* (MO); Bottoms, P.O. Malvern, Hot Springs Co., *D. Delmaree 14502* (MO); Brazil, *D. Delmaree 10971* (MO); Bruton,* J.M. Greenman 4299*, (MO); Flat woods, P.O. Star City, *D. Delmaree 16748* (MO); Lake City, *D. Delmaree 6945* (MO); Lookout Mountain. Near the line between Tennessee and Georgia which crosses the mountain, *J.R. Churchill s.n*. (MO); Monticello PO, *D. Delmaree 1661* (MO); Near Nogo, *G.M. Merrill 61* (MO); Nogo, *G.M. Merrill 168* (MO); Ponca, *L. Hubricht 1357* (MO); Rush, Marion County, along streams,* E.J. Palmer 6031* (MO); Springs National Park, *L.H. Pammel 189* (MO); Westfork, *E.J. Palmer 8223* (MO); Woods along White River, near Calico Rock, *E.J. Palmer 35546* (MO); Yell County: Bridge over the Petit Jean River, 2.6 miles north of Ola on State Hwy., *T.G. Lammers 8304* (F); **Conneticut**: Driftway Lane, *F.C. Seymour 20577* (MO); Woods, Tokeneke, near Darien, *R. C. Ward s.n*. (MO); **Florida**: Eglin Air Force Base; 11.7 mi east of Florida State Route 285 on Air Force Base Route 213, *J.S. Miller 9554* (MO); Gainesville and vicinity of Lake Alice, *T.B. Croat 25056* (MO); Near Jacksonville, *A.H. Curtiss 4530* (MO); Hummock land, vicinity of Eustis, Lake County, *G.V. Nash 860* (MO); Swales along Rt. 17, N of Yulee near Goodbread Circle, *C.F. Reed 102948* (MO); Tall Timbers Research Station on S-12 north of Talahassee, *D.A. Breil 376* (MO); Woods near Oak Hill, Volusia Co., *C.F. Reed 35659* (MO); Woods, Rt. 41, 2 mi N of Brooksville, *C.F. Reed 101308* (MO); **Georgia**: 1/2 mi S of Brewton, *G.L. Bracewell 42* (MO); 2.4 mi E of Ailey on US 280, *J.C. Solomon 2070* (MO); Alapha River swamp, 6 mi S of Stockton, *J. Norsworthy* (MO); Edge of Damp woods, Leslie, *R.M. Harper 1391* (MO); Farm Woods, Rt. 301 just N of Ogeeche River, *C.F. Reed 116746* (MO); Flood plain and banks of Ty Creek, *W.R. Faircloth 3374* (MO); Hammock area (elevated terrain) surrounded by low pinelands on the E side of Moody AFB Reservation, *W.R. Faircloth 3709* (MO); **Illinois**: 2.9 mi S of Gorham Rd. on ILL 3, then 3.9 mi E on Turkey Bayou Rd., bank of the big muddy river, *J.C. Solomon 3719* (MO); Fountain Bluff, mesic woods along summit roadside, *R. Carlson s.n*. (MO); Horseshoe Island, *G.N. Jones 12054* (MO); Mound city, *E.J. Palmer 14826* (MO); Near Mississippi River, Chester, *E.J. Palmer 44553* (MO); Tunnel Hill, *E.J. Palmer 15227* (MO); **Indiana**: Awensvillle, *C.F. Reed 3213* (MO); Near Arlington, *C.F. Reed 3494* (MO); Brandywine, *C.F. Reed 2840* (MO); Calvert Co., *C.F. Reed 2463* (MO); Damp woods along River R. near Herald Beach, *C.F. Reed 30915* (MO); David Hill Park, Balto, *W.N. Lee 6* (MO); Double Rock Park, Parkerville, *L.E. Schauer s.n*. (MO); Flats Region, Guthrie Memorial Tract, *R.C. Friesner 10161* (MO); Indian Head, *C.F. Reed 3079* (MO); Lower Marlboro, *C.F. Reed 4829* (MO); marches just south of Curtes Bay, Brandens Shore, *C.F. Reed 31066* (MO); Middle River, *C.F. Reed 3094* (MO); Oak-maple woods, Rt. 313, 1 mi N of Mossey, *C.F. Reed 127985* (MO); on Rantan Formation, Elk Neck State Park at Rogus Harbor Boating facility cutoff, *C.F. Reed 126623* (MO); Pt. Lookout .527, St. M., *C.F. Reed 2476* (MO); Shiloh, *C.F. Reed 409* (MO); Swampy woods E of Patiewment, S of Upper Marlboro, *C.F. Reed 22817* (MO); Swampy woods near Pomonkey, *C.F. Reed 23414* (MO); Wicomico, *C.F. Reed 2533* (MO); woods along road to Southland Bog, *C.F. Reed 125997* (MO); woods just north of East Riverside, *C.F. Reed 29353* (MO); Woods Point Park, Bush R., *C.F. Reed 128078* (MO); woods south of Drum Point, *C.F. Reed 29290* (MO); Warwick, between Rt. 282 and Rt. 301, *C.F. Reed 136283* (MO); **Kentucky**: 1 mile north of Cogswell, Rowan County, wet ditches, *C.F. Reed s.n*. (MO); 1 mile north of Cogswell, Rowan County, wet ditches, *C.F. Reed 13437* (MO); along Ison Creek, on igneaous mica-perodotite dike west of Stephens, *C.F. Reed 115816* (MO); along Axley Branck, Morehead, *H. Williston 111* (MO); along Maxon Rd., just w of Concord, off Rt. 60, *C.F. Reed 110706* (MO); Bowling Green, *S.F. Price s.n*. (MO); Georgetown, *C.F. Reed 81477* (MO); Low woodlands, Rte. 68, near Erie, *C.F. Reed 129641* (MO); Near Reed, Rt. 60, *C.F. Reed 117576* (MO); Near Wago, Rt. 90. 3 mi W of Albany, *C.F. Reed 52888* (MO); Redd Hallow, L.B.L., 0.5 mi W of Kentucky 94, *K. Hutchens 12* (MO); Rt. 42, Skylight, *C.F. Reed 44907* (MO); Robey’s Swamp, 3 mi NW of Franklin, *C.F. Reed 82311* (MO); Rt. 45, 1.5 mi NE of Water Valley, *C.F. Reed 52801* (MO); Rt. 470, Larue Co., near Buffalo, *C.F. Reed 45066* (MO); Woods at Lovely, *C.F. Reed 75623* (MO); Woods, Diohmans Springs, 7 mi NW of Barbourville, *C.F. Reed 7176* (MO); Woods near Goddard, Fleming Co., *C.F. Reed 10446* (MO); **Louisiana**: 15 ft. from University Lake along Lousiana 42, S of Baton Rouge, *K. Sijam s.n*. (MO); 2 miles north of Gorum, Kisatchie Wood, Natchitiches Parish, *J. Ewan 17630* (MO); 6 mi West of Ruston on Lousiana 507, *P. Lohman 31* (MO); Cemetery, waste area and neighboring woodlands, *J.K. Parrott 427* (MO); Cypress Creek bottom, two miles N of Vienna, *G. Fischer 20* (MO); just north of D’Arbonne Fire Tower, *P. Both 315* (MO); Ponchatoula, Williams Lumber, (MO); **Mississipi**: Meramec State Park. Cambell Hollow, *D. Dress 4* (MO); Natchez, *F. Shimak s.n*. (MO). Ocean Springs, Shekan, J., (MO); **Missouri**: at base of limestone bluffs alongs Frederick Pork near “The Narrows”, west of Calm, *J.A. Steyermark 18919* (MO); base of Black Mtn., *J.A. Steyermark 21077* (MO); base of wooded lime slopes along spring branch of Williams spring on east side of current river, T25N, R1E, sect. 34, SW of Grandin, *J.A. Steyermark 11853* (MO); Bilsted, Gum near Illmo, *A. Christ s.n*. (MO); Campbell Mts., *B.F. Bush 227* (MO); Ca. 8 mi NE of Salem (T35 R4W S33 S2 SE4), Upper Ozark Section of Ozark Natural Division. Indian Trail conservation Area, *T. Smith 3500* (MO); Columbia, near corner of Paquin an Waugh Streets, *C. Dietrich 265* (MO); Kirkwood, *F. Comte 437* (MO); low ground area in valley Blue Spring Itol, T27 N, R7 E sect. 20, 2 mi E of Chaonia, *J.A. Steyermark 6313* (MO); lowland woods between Menfro and Belgique, 2 mi southwest of Belgique, *J.A. Steyermark 14027* (MO); on a siding of the Terminal Railroad Association , *V. Muehlenbach 3361* (MO); Pleasant Grove, *B.F. Bush 258* (MO), *K.K. Mackenzie 421* (MO); Poplar Bluffs, *G. Letterman s.n*. (MO), *H. Eggert s.n*. (MO); rich swampy woods on elevated portion, *J.A. Steyermark 8662* (MO); Timber Tract, 10 mi southwest of Wolf Island; Low woods along mud creek, T26N, R7E, sect. 20, 2 mi northwest of Rombauer, *J.A. Steyermark 11294* (MO); Williamsville, *E.J. Palmer 4805* (MO); Woods, Butler County, *B.F. Bush 3721* (MO); **North Carolina**: Dry open woods east of Chapel Hill off 15-501, *J.P. May 95* (MO); near Sisk’s house, foot of Table rock, *S.K. Small 292* (MO); Swamps, Bladen Country, Biltmore , 4178, (MO); Open marshy area, Durham, *C. Henninger s.n*. (MO); Woods just S of Grandy, Currituk Co., *C.F. Reed 41634* (MO); **Oklahoma**: Broken Bow, *E.J. Palmer 10496* (MO); growing on flat ground in valley, *H. Newton 94* (MO); near Page, *G.W. Stevens 2634* (MO); wet sandy loam soil in a wood, 4 mi SW of Broken Bow, *R. Stratton 585* (MO); **Pennsylvania**: N of Boulevard, along Philadelphia-Trenton border of P.R.R., Cedar Grove, *J.W. Adams 177* (MO); **South Carolina**: 1 mile north of Bloomfield, low pasture, beside Hwy 25, *A. Chandler 2047* (MO); Anderson, *J. Davis s.n*. (MO); McKinney Spring, *J. Davis s.n*. (MO); Simpson’s Mill near Anderson, *J. Davis s.n*. (MO); Upper coastal plain, sandy soil, *M.L. Conrad 11171* (MO): **Tennessee**: Hancock-Claiborne County line, Damp cove, *Vogenberger 8066* (MO); Knoxville, *A. Ruth s.n*. (MO); Aumville (?), *A. Ruth 271* (MO); Nashville 12 mi west on River Rd., *E. Quaterman 1036* (MO); Near Memphis, *E.J. Palmer 17516* (MO); Near Shepherd, *E.J. Palmer 17478* (MO); **Texa**s: 2nd Bottom, *E.D. Marshall 8711* (MO); Big Thicket National Preserve. Lance Rosier Unit. Ca., *J. Stone 3096* (MO); Houston, *B.F. Bush 16, 22* (MO); Livingston, Polk county, *E.J. Palmer 5235* (MO); **West Virginia**: Banks of James River, near Richmond, *J.R. Churchill s.n*. (MO); Camden Ave., near Ritchie St., Buchanon, *G.B. Rossbach s.n*. (MO); Carrollton, Isle of Wight Co, *C.F. Reed 102408* (MO); Huntington, *Williams 306* (MO); Miles Creek, Rt. 58, 6 mi S of South hill, *C.F. Reed 53398* (MO); Paducah, McCracken County, *E.J. Palmer 17884* (MO); Pigeon creek near Enons School, Mingo County, *E.E. Berkley 966* (MO); Rt. 617 off Rt. 205, NE of Edgehill, *C.F. Reed 130382* (MO); woods near Norfolk, *F. Blanchard* vicinity of Norfolk, *M.C. Jensen s.n*. (MO); Walls Run Creek at Rt. 10 near Rt. 609, *C.F. Reed 89698* (MO); woods south of Suffolk, *C.F. Reed 8946* (MO); *s.n*. (MO); woods, 8 mi SW of South Hill, Rt. 58, Mecklenburg Co., *C.F. Reed 53404* (MO); woods along Rt. 14, Mattaponi, King and Queen Co., C.*F. Reed 45994* (MO); woods just E of Pohick Church, on Old Colchester Rd., *C.F. Reed 103177* (MO); woods just W of Camplain P.O., Rt. 17, *C.F. Reed 100957* (MO).

#### 15. Liquidambar yunnanensis

(Rehder & Wilson) Ickert-Bond & J. Wen comb. nov.

urn:lsid:ipni.org:names:77134714-1

http://species-id.net/wiki/Liquidambar_yunnanensis

Basionym: *Altingia yunnanensis* Rehder & Wilson, in Sargent Pl. Wilson. I: 422 (1913). TYPE. CHINA: Yunnan: Mengtze, A. *Henry 10395* (Holotype: A [A0043393!]; isotypes: K [H2007/01764!], NY [NY00356723!]).

##### Distribution.

China (Yunnan), Vietnam. **Representative specimens examined.** China, Yunnan, Mengtze, A. *Henry 11082* (A). Vietnam: **Lao Cai**: road to O’Qui Ho, SaPa, *Institute Bot. Yunnanica s.n*. (HN); **Cao Bang**: Cao Son, *Poilane 19024* (P000317376!).

### Doubtful names

1. *Altingia indochinensis* H. T. Chang, Acta Sci. Nat. Univ. Sunyatseni, 1961 (4): 53 (1961). Type: Indochina, without locality, *M. Poilane 2000*. We have not located the type specimen.

According to Chang (1961), this species is closely allied to *Altingia gracilipes* Hemsl. and its variety *Altingia gracilipes* Hemsl. var. *serrulata* Tutch., but differs from them by longer, oblanceolate or oblong leaves which are cuneate at the base, more robust petioles, and multiflorous heads.

### Excluded names

1. [Araucariaceae *Altingia cunninghamii* in Hort. Brit. [Loud.] 403 (1830).

Notes: =*Araucaria cunninghamii*]

*Altingia cunninghamii* J. Ross , Hobart Town Almanack (1835) 66.

Remarks. Given as a name without description by G.Don in J.C. Loudon, Hort. Brit. (1830) 403. Not in Index Kewensis. “lately discovered by Mr. Ronald Gunn, at the falls of the river Meander and also by Mr. J.W. Scott, the collector on the banks of the Huon”.

2. [Araucariaceae *Altingia excelsa* in Hort. Brit. [Loud.] 403 (1830).

Notes: =*Araucaria excelsa*]

## Supplementary Material

XML Treatment for Liquidambar

XML Treatment for Liquidambar acalycina

XML Treatment for Liquidambar cambodiana

XML Treatment for Liquidambar caudata

XML Treatment for Liquidambar chinensis

XML Treatment for Liquidambar chingii

XML Treatment for Liquidambar excelsa

XML Treatment for Liquidambar formosana

XML Treatment for Liquidambar gracilipes

XML Treatment for Liquidambar multinervis

XML Treatment for Liquidambar obovata

XML Treatment for Liquidambar orientalis

XML Treatment for Liquidambar poilanei

XML Treatment for Liquidambar siamensis

XML Treatment for Liquidambar styraciflua

XML Treatment for Liquidambar yunnanensis

## Appendix

**Index to Numbered collections:**

Adams, J.W. 177 (styraciflua)

Aksoy 5203 (orientalis), 5204 (orientalis)

Allen, P.H. 11181 (styraciflua), 7265 (styraciflua)

Amador, G. 172 (styraciflua)

Andersen, J.S. 2081 (orientalis)

Araquistain, M. 3538 (styraciflua)

Arp, G.K. 4186 (styraciflua)

Atwood, J.T. 6816 (styraciflua), 9 (styraciflua)

Avendaño, R. 277 (styraciflua)

Bailey, L.H. s.n. (formosana)

Bakhuizen 6372 (excelsa)

Balansa, B. 1156 (formosana)

Balgooy, M.J. v. 2912 (excelsa)

Bartholomew, B. 1758 (acalycina), 1950 (acalycina), 773 (formosana)

Beaman, J.H. 5148 (styraciflua), 5970(styraciflua), 6276(styraciflua)

Bejaud, M. 877 (siamensis)

Berkley, E.E. 966 (styraciflua)

Blanchard, F. s.n. (styraciflua)

Bodinier, E. 1042 (chinensis)

Bogle, A.L. 1561 (orientalis)

Bohs, L. 1798 (styraciflua)

Boratynska, K. 15 (orientalis), 164 (orientalis), et al. 15 (orientalis)

Both, P. 315 (styraciflua)

Botteri 860 (styraciflua)

Boufford, D.E. et al. 37528 (formosana)

Bourgeau, M. 24 (styraciflua)

Boutin, F. 3480 (styraciflua), 3485 (styraciflua), 3494 (styraciflua)

Bracewell, G.L. 42(styraciflua)

Breedlove, D.E. 6886 (styraciflua), 21762 (styraciflua), 34365 (styraciflua)

Breil, D.A. 376 (styraciflua)

Brillet, M. 19 (chinensis)

Burger, W. 26 (styraciflua)

Bush, B.F. 16, 22 (styraciflua), 227 (styraciflua), 2425 (styraciflua), 258 (styraciflua), 3721 (styraciflua)

Calderon, S. 1928 (styraciflua)

Calzada, J.I. 5246 (styraciflua), 5250 (styraciflua), 539 (styraciflua)

Canton Christian College Hebrarium 7700 (formosana)

Carles, W.R. s.n. (formosana)

Carlson, M.C. 608 (styraciflua), 757 (styraciflua)

Carlson, R. s.n. (styraciflua)

Castillo, C.G. 1350 (styraciflua), 1803 (styraciflua)

Champion 325 (chinensis)

Chandler, A. 2047 (styraciflua)

Chang, H.T. 4701 (acalycina)

Chang, S.Y. 4837 (caudata)

Chen, H. 1272 (gracilipes)

Chen, S.-Q. 14715 (acalycina), 3346 (acalycina)

Cheo, T.Y. 1149 (formosana)

Cheun, Y.R. s.n. (formosana)

Chevalier, A. 2964 (formosana), 37471 (formosana)

Chevez, S.Y. 119 (styraciflua)

Chiao, C.Y. 14238 (formosana)

Chien, S.S. 1019 (formosana)

China Vietnam team 8462 (poilanei)

Ching, R.-C. 1578 (formosana), 2199 (gracilipes), 2244 (chingii), 4823 (formosana), 4823 (formosana), 7937 (chinensis)

Chiu, P.L. 1072 (gracilipes), 1470 (gracilipes), Chiu 961 (acalycina)

Chow, H.-C. 730 (formosana)

Christ, A. s.n. (styraciflua)

Chun, N.K. 42718 (gracilipes), 44321 (obovata)

Chung, H.H. 1006 (formosana), 2132 (formosana), 2901 (formosana), 40 (formosana), 866 (formosana)

Churchill, J.R. s.n. (styraciflua)

Clemens, J. 3388 (siamensis)

Comte, F. 437 (styraciflua)

Conrad, J. 3262 (styraciflua)

Conrad, M.L. 11171 (styraciflua)

Croat, T.B. 25056 (styraciflua)

Cuong, N.M. 93 (formosana)

Curtiss, A.H. 4530 (styraciflua)

Dai, T.L. 103616 (excelsa) after Curtiss

Daniel, T.F. 9628 (styraciflua)

Danish Bot. Trans-Asia Expedition III No. 2081 (orientalis)

Davi, P.H. 13474 (orientalis)

Davidse, G. 34570 (styraciflua)

Davis 40317 (orientalis)

Davis, J. s.n. (styraciflua )

De Voogd 1772 (excelsa)

Delmaree, D. 10971 (styraciflua), 6945 (styraciflua), 14502 (styraciflua), 1661 (styraciflua), 16748 (styraciflua)

Deng, L. 3116 (formosana), 5031 (siamensis), 5438 (gracilipes), 4331 (gracilipes), Dong 4685 (gracilipes)

Deng, L. 3549 (chingii), 6681 (chingii)

Deramu, R. 1043 (styraciflua)

Dietrich, C. 265 (styraciflua)

Donnat 38180 (formosana)

Dress, D. 4 (styraciflua)

Dumat 414 (excelsa)

Dunn, S.T. 2682 (gracilipes), Dunn s.n. (gracilipes)

Earle, F.S. s.n. (styraciflua)

Eggert, H. s.n. (styraciflua)

Endert, T.H. 1068 (excelsa)

Ewan, J. 17630 (styraciflua)

Exped. of Guizhou Academy of Science 76536 (chingii)

Faircloth, W.R. 3374 (styraciflua), 3709 (styraciflua)

Fan, C.S. 280 (formosana), 75 (formosana)

Fang, W.P. 811 (formosana)

Fassett, N.C. 28302 (styraciflua)

Faurie, A. 279 (formosana)

Faurie, U. 45 (formosana)

Feng, Z. 36293 (gracilipes)

Field Museum 373260 (excelsa)

Fiori, A. 130 (orientalis), 230 (orientalis), 231 (orientalis)

Fischer, G. 20 (styraciflua)

Fleury, F. 30170 (formosana, 37961 (formosana)

Forbes, H.O. 1201 (excelsa)

Forrest, G. 18414 (excelsa), 8763 (excelsa)

Friesner, R.C. 10161 (styraciflua)

Fu, K.-J. 5799 (formosana)

Fujian Forestry Coll. 41 (gracilipes)

Fujian Team 5567 (gracilipes)

Fung, H. 20245 (formosana)

FUS expedition 53260 (chingii)

Gentry, A. 32417 (styraciflua)

Gomez-Pompa 5382 (styraciflua)

Goner 9145 (orientalis)

Gonzalez, L.G. 233(styraciflua)

Greenman, J.M. 4299 (styraciflua)

Gressitt, J.L. 2415 (acalycina)

Grieson, A. 3581 (excelsa)

Griffith 286 (excelsa), 3380 (excelsa)

Grijalva, A. 250 (styraciflua)

Handel- Manzetti, H. 185 (formosana)

Hangzhou Botanial Garden 7303 (caudata)

Harder, D.K. et al. 4180 (formosana)

Hardial 601 (siamensis)

Harmand 13007 (formosana)

Harper, R.M. 1391 (styraciflua)

Hawkins, T. 176 (styraciflua)

He, G. 4690 (gracilipes), 5479 (gracilipes), 6155 (gracilipes)

He, G.-S. 6179 (formosana)

Henninger, C. s.n. (styraciflua)

Henry, A. 10395 (yunnanensis), 11082 (yunnanensis), 425 (formosana), 5218 (formosana), 52181 (acalycina), 7630 (formosana)

Hernandez, M. 1610 (styraciflua), 448 (styraciflua), 558 (styraciflua), 5630 (styraciflua), 5790 (styraciflua), 6912 (styraciflua), 1499 (styraciflua)

Hers, J. 2264 (formosana), 608 (formosana)

How, F.C. 20124 (formosana), 70369 (obovata)

Howard, R. 627 (styraciflua)

Hu, S.Y. 20953 (formosana), 9984 (gracilipes)

Hu, S.Y. & K.H. Yung s.n. (formosana)

Huang, Z. 44074 (chingii)

Hubricht, L. 1357 (styraciflua)

Hughes, C.E. 330(styraciflua)

Hutchens, K. 12 (styraciflua)

Ickert-Bond, S. 1260 (formosana), 1261 (chinensis), 1266 (gracilipes), 1272 (gracilipes), 1274 (chinensis), 1280 (siamensis), 1290 (formosana), 1290, 1291 (formosana), 1291 (formosana), 1296 (poilanei), 1305 (formosana), 1307 (chingii), 1308 (chingii), 1309 (formosana), 1321 (formosana), 1324 (formosana), 1326 (formosana), 1327 (formosana), 1328 (formosana), 1310 (chingii), 1311 (chingii)

Iltis, H.H. 942 (styraciflua)

Institute Bot. Yunnanica s.n. (yunnanensis)

Jayaweera, D.M.A. 1617 (excelsa)

Jensen, M.C. s.n. (styraciflua)

Jones, G.N. 12054 (styraciflua)

Keenan, J. 1407 (excelsa), 1407 (siamensis), 1528 (excelsa), 1940 (excelsa), 3679 (excelsa)

Keng, Y.L. 316 (gracilipes)

Kerr 5110 (siamensis)

Khan 45 (orientalis)

Kin Dao, N. 182 (siamensis)

Kingdon-Ward, F. 20761 (excelsa)

Ko, S.P. 56024 (chinensis), 534488 (chingii)

Koordes 15754 (siamensis)

Kyoto University 41655 (excelsa)

Lammers, T.G. 8304 (styraciflua)

Lau, S. K.1645 (formosana), 4809 (formosana), 25962 (formosana)

Lau, T.W. 98 (gracilipes)

Lau, Y.S. 206 (chinensis)

Lee, W.N. 6 (styraciflua)

Lei, C.I. 355 (formosana)

Letterman, G. s.n. (styraciflua)

Levine, C.O. 1572 (formosana), 1731 (formosana), 3158 (chinensis), 3302 (formosana)

Li, X. 200701 (gracilipes), 200921 (gracilipes), 202545 (gracilipes)

Li, Z. 1040 (gracilipes), 1162 (gracilipes), 4972 (obovata), 963 (gracilipes)

Li, Z.-X. 3841 (formosana)

Liang 3685 (obovata)

Liang, H.Y. 61283 (chingii), 62594 (obovata), 64371 (obovata), 64734 (obovata), 69714 (chinensis),

Liang, X.-R. 62343 (formosana)

Liebmann, F.M. 3052 (styraciflua)

Liesner, R.L. 26609 (styraciflua)

Lignan Team 12347 (formosana)

Lin, Y.-R. 516 (formosana)

Ling, Y. 2295 (gracilipes), 4522 (chingii)

Liu, L.-H. 542 (acalycina)

Liu, L.L. et al. s.n. (formosana)

Liu, Y. 242 (gracilipes), 90242 (gracilipes)

Lo, L.T. 629 (gracilipes)

Loc, P.K. et al. 1704 (formosana)

Loerzing, J.A. 17368 (excelsa)

Lohman, P. 31 (styraciflua)

Lopez Luna, R. 27(styraciflua)

M.L. Nie 91192 (acalycina)

M.T. Sin & W.K. Woo s.n. (gracilipes)

Mackenzie, K.K. 421 (styraciflua)

Manriquez, G. 2339 (styraciflua)

Mao, Z.G. 10231 (gracilipes)

Mao, Z.G. 10237 (caudata)

Marquez, R.W. 997 (styraciflua)

Marshall, E.D. 8711 (styraciflua)

Martinez, S.E. 24118(styraciflua), 3872(styraciflua), 3972 (styraciflua)

Martinez, V.M. 142, 454 (styraciflua)

Matuda, E. 1157 (styraciflua)

May, J.P. 95 (styraciflua)

Maxwell, J.F. s.n (siamensis)

McDonald & Ismail 4966 (excelsa)

Meijer, Willem s.n. (excelsa)

Mejia, D. 357 (styraciflua), 402 (styraciflua)

Mendez Ton, A. 6087 (styraciflua)

Merrill, E. 1731 (formosana)

Merrill, G.M. 168 (styraciflua), Merrill 61 (styraciflua)

Metcalf, F.T. & T.C. Chang 877 (formosana)

Metcalfe, F.P. 9360 (formosana)

Meyer, F.G. 2799 (styraciflua)

Miller, J.S. 9554 (styraciflua)

Moera-Laboch For. Serv, Neth. Ind. 18066 (excelsa)

Molina, R.A. 10807 (styraciflua), 11034 (styraciflua), 11342 (styraciflua), 11454 (styraciflua), 11940 (styraciflua), 12578 (styraciflua), 13755(styraciflua), 20435 (styraciflua), 22235 (styraciflua), 6127 (styraciflua), 7254 (styraciflua), 8430 (styraciflua), 2834 (styraciflua), 983 (styraciflua)

Monro, A.K. 2626 (styraciflua)

Monyrak, M. 10 (siamensis)

Moore, H.E. 1925 (styraciflua), 1995 (styraciflua), 2176 (styraciflua)

Moreno, P.P. 22766 (styraciflua), 25910 (styraciflua)

Muehlenbach, V. 3361 (styraciflua)

Murray, E. 1020 (orientalis)

N.K. Chun 42718 (gracilipes)

Nash, G.V. 860 (styraciflua)

National Geographic Society Hainan Expedition 46 (chingii)

Nee, M. 22484 (styraciflua), 23457 (styraciflua), 26005 (styraciflua), 26198 (styraciflua), 26295 (styraciflua), 26393 (styraciflua), 26885 (styraciflua), 28898 (styraciflua), 29442, 29444 (styraciflua), 29689 (styraciflua), 29700 (styraciflua)

Neil, D.A. s.n. (styraciflua)

Newton, H. 94 (styraciflua)

Ngoc Kinh, H. 169 (chingii)

NGS Hainan Expedition 46 (chingii)

Norris, D.H. 17397(styraciflua)

Norsworthy, J. (styraciflua)

Oersted, A.S. 3050 (styraciflua)

Oldham, R. s.n. (formosana)

Ortega, O.R. 1234 (styraciflua)

Ortega, R. 837 (styraciflua)

Palmer, E.J. 10496 (styraciflua), 14826 (styraciflua), 15227 (styraciflua), 17478 (styraciflua), 17516 (styraciflua), 17884 (styraciflua), 35546 (styraciflua), 44553 (styraciflua), 4805 (styraciflua), 6031 (styraciflua), 8223 (styraciflua), 53823 (styraciflua)

Palmer, E.J. 5235 (styraciflua)

Pammel, L.H. 189 (styraciflua)

Parrott, J.K. 427 (styraciflua)

Pena, M. 1046 (styraciflua)

Perino, C.H. 3262(styraciflua)

Petelot. A. 2332 (chinensis), 5747 (formosana)

Phuong, T. Tien 2539 (formosana)

Phuong 4647 (formosana)

Phuong, V. Xuan 586 (siamensis)

Pierre, L. s.n. (siamensis)

Ping, K. 1597 (formosana)

Poilane, M. 19024 (yunnanensis), 12844 (poilanei), 1317 (formosana), 13500 (siamensis), 1610 (formosana), 2000 (siamensis), 22153 (siamensis), 3228 (siamensis), 9938 (siamensis), 263 (cambodiana), s.n. (cambodiana)

Polunin, G. 14923 (orientalis)

Prain 769 (excelsa)

Price, S.F. s.n. (styraciflua)

Pringle, C.G. 7754 (styraciflua)

Proctor, G.R. 25212 (styraciflua)

Quaterman, E. 1036 (styraciflua)

Raven, P.H. 19784 (styraciflua), 26323 (styraciflua)

Rechinger, K.H. 8550 (orientalis)

Reed, C.F. 100957 (styraciflua), 10130 (styraciflua), 102408 (styraciflua), 102737 (styraciflua), 10294 (styraciflua), 103177 (styraciflua), 103825 (styraciflua), 10446 (styraciflua), 110706 (styraciflua), 115816 (styraciflua), 116746 (styraciflua), 117576 (styraciflua), 125997 (styraciflua), 126623(styraciflua), 12798(styraciflua), 12807(styraciflua), 129641 (styraciflua), 130382 (styraciflua), 13437 (styraciflua), 136283(styraciflua), 149022 (styraciflua), 22817 (styraciflua), 23414 (styraciflua), 2463 (styraciflua), 2476 (styraciflua), 2533 (styraciflua), 2840 (styraciflua), 29290 (styraciflua), 29353(styraciflua), 3079 (styraciflua), 30915 (styraciflua), 3094 (styraciflua), 31066 (styraciflua), 3213 (styraciflua), 3494 (styraciflua), 35659 (styraciflua), 41634 (styraciflua), 44907(styraciflua), 45066 (styraciflua), 45994 (styraciflua)4829 (styraciflua), 52801 (styraciflua), 52801(styraciflua), 52888 (styraciflua), 53398 (styraciflua), 53404 (styraciflua), 7176 (styraciflua), 75623 (styraciflua), 81477 (styraciflua), 82311 (styraciflua), 8946 (styraciflua), 89698 (styraciflua), s.n. (styraciflua)

Regalado, J.C. et al. HLF4449 (siamensis)

Renfrow, J. 16 (styraciflua)

Reyes Rivera, J. 257 (styraciflua)

Rivera, R.J. 907 (styraciflua)

Rock, J.F. 2768 (excelsa), Rock 7174 (excelsa)

Rodriguez, V.J. 1883 (styraciflua), 2840 (styraciflua)

Rossbach, G.B. s.n. (styraciflua)

Rubio, A. 63 (styraciflua)

Rueda, R. 16311 (styraciflua)

Ruth, A. 271 (styraciflua), s.n. (styraciflua)

S.Y. Hu 20064 (formosana)

SABE 1950 (acalycina)

Saito, S. 8371 (formosana),

SAYTBET 45208 (formosana), 45272 (formosana)

Schauer, L.E. s.n. (styraciflua)

Seigler, D. 3601 (styraciflua)

Seler, E., 3024 (styraciflua)

Seymour, F.C. 205 (styraciflua)

Shekan, J. s.n. (styraciflua)

Shimak, F. s.n. (styraciflua)

Sijam s.n. (styraciflua)

Skutch, A.F. 1739 (styraciflua)

Small, S.K. 292 (styraciflua)

Smith, C.L. 1778 (styraciflua)

Smith, J.D. 1855 (styraciflua)

Smith, T. 3500 (styraciflua)

Smitinand, T. 10848 (excelsa)

Soejarto, D.D. 11399 (formosana), DDS 13 (siamensis)

Solomon, J.C. 2070 (styraciflua, 3719 (styraciflua)

Song, X.H. 1348 (chinensis)

Sousa, M. 3433 (styraciflua)

Sozan, T. 13490 (formosana)

Standley, P.C. 10026 (styraciflua), 13708 (styraciflua), 23747(styraciflua), 56158 (styraciflua), 6497 (styraciflua), 70309 (styraciflua), 887 (styraciflua), Tsoong 256 (multinervis),

Stevens W.D. s.n. (styraciflua), 11510 (styraciflua), 15074 (styraciflua), 17665 (styraciflua), 18095 (styraciflua), 21054 (styraciflua)

Stevens, G.W. 2634 (styraciflua), 3248(styraciflua)

Steward, A.N. 107 (formosana), 1075 (formosana), 29 (formosana), 442 (formosana)

Steyermark, J.A. 29732 (styraciflua), 42409 (styraciflua), 49713 (styraciflua), 5172 (styraciflua), 11294 (styraciflua), 11853 (styraciflua), 18919 (styraciflua), 21077 (styraciflua), 6313 (styraciflua), 8662 (styraciflua)

Stone, J. 3096 (styraciflua)

Stratton, R. 585 (styraciflua)

Sun, S.C. 1302 (formosana)

Taam, Y.M. 779 (formosana)

Tan, P.X. 57605 (formosana), 58681 (chingii)

Tang Khoi, N. 420, 421 (formosana)

Tang, C.C. 4655 (formosana)

Tang, H.C. 1473 (formosana)

Tenorio, L.P. 14900 (styraciflua), 5329 (styraciflua)

Thai Van Trung 108 (siamensis)

Thorne, R.F. 40034 (styraciflua)

Tien Ba, N. 110 (siamensis)

Tomlin, S. 176 (styraciflua)

Torres, R. 2541 (styraciflua)

Tsai, H.T. 61528 (excelsa)

Tsang, W.T. 20128 (chinensis), 20927 (formosana), 21514 (gracilipes), 21697 (gracilipes), 21817 (formosana), 22189 (chinensis), 22788 (chinensis), 23613 (formosana), 25942 (chinensis), 28068 (formosana), 28544 (chinensis), 20927 (formosana), 6412 (styraciflua)

Tsang, W.T. 21697 (gracilipes), 23613 (formosana)

Tsiang, Y. 6677 (siamensis)

Tso 1803 (formosana)

Tso. C.L. 21049 (chinensis)

Tsoong, T.S. 8197 (formosana), 81972 (formosana)

Tsui, T.M. 417 (formosana), 516 (formosana), 660 (formosana)

Tucker, J. M. 1209 (styraciflua)

Tue, H. 523 (siamensis)

Tuerckheim, H. 1804 (styraciflua)

Tutcher, W. J. s.n. (gracilipes)

van Beusekom, C. F. 4102 (siamensis)

van de Koppel 3299 (excelsa)

Vargaz Ruiz, E. 44, 124 (styraciflua)

Vazquez, T.V. 260 (styraciflua), 286 (styraciflua)

Ventura, F.A. 1925 (styraciflua)

Vidal, J.E. 4461 (siamensis)

Vogenberger 8066 (styraciflua)

Wan, X. 153 (gracilipes)

Wang, C. 31824 (formosana), 31828 (chinensis), 35099 (obovata), 35691 (obovata), 35897 (obovata), 36153 (obovata), 36638 (obovata), 37057 (gracilipes), 39334 (chinensis), 44102 (acalycina)

Wang, Y.-J. 56484 (gracilipes)

Warburg, O. 9810 (formosana)

Ward, R.C. s.n. (styraciflua)

Webster, G.L. 17747 (styraciflua)

Wei, Z. 120217 (gracilipes), 120579 (gracilipes)

Williams 306 (styraciflua)

Williams, L. 12190 (styraciflua)

Williams, L.O. 17113 (styraciflua), 28004 (styraciflua), 23388 (styraciflua)

Williston, H. 111 (styraciflua)

Wilson, E.H. 10031 (formosana), 1628 (formosana), 218 (formosana), 513 (acalycina), 795 (formosana), 1639 (formosana), 513 (formosana),

Wu, Z. 85062 (chingii)

Wuyi Shan Team 80-225 (acalycina)

Xiong, B. 20927 (formosana), 2922 (formosana), 5706 (formosana)

Yao, K. 11486 (acalycina)

Yao, K. 11561 (formosana)

Yao, K. 8505 (formosana)

Ye, C.-L. 9689 (formosana)

Yin, H.L. 2031 (formosana)

Yu, B. 103106 (gracilipes)

Yu. T.T. 229 (formosana)

Yuncker, T.G. 5830 (styraciflua), 8776 (styraciflua)

Zeng, H. 72101 (gracilipes)

Zhang, Z.-Y. 14271 (formosana)

Zhong, Y. 4321 (obovata)

Zola, M.G. 500 (styraciflua), 616 (styraciflua), 651 (styraciflua), 697 (styraciflua), 746 (styraciflua)

Zou, H.-Y. 140 (formosana), 151 (gracilipes), Zou 1984 (acalycina), 307 (chinensis), 761 (chinensis), 762 (gracilipes), 79 (gracilipes)

Zuo, J.B. 356 (acalycina)
